# Mitochondrial health is enhanced in rats with higher vs. lower intrinsic exercise capacity and extended lifespan

**DOI:** 10.1038/s41514-020-00054-3

**Published:** 2021-01-04

**Authors:** Miguel A. Aon, Sonia Cortassa, Magdalena Juhaszova, José A. González-Reyes, Miguel Calvo-Rubio, José M. Villalba, Andrew D. Lachance, Bruce D. Ziman, Sarah J. Mitchell, Kelsey N. Murt, Jessie E. C. Axsom, Irene Alfaras, Steven L. Britton, Lauren G. Koch, Rafael de Cabo, Edward G. Lakatta, Steven J. Sollott

**Affiliations:** 1 Laboratory of Cardiovascular Science, National Institute on Aging, NIH, Baltimore, MD 21224 USA; 2 Experimental Gerontology Section, Translational Gerontology Branch, National Institute on Aging, NIH, Baltimore, MD 21224 USA; 3 Departamento de Biología Celular, Fisiología e Inmunología, Campus de Excelencia Internacional Agroalimentario, ceiA3, Universidad de Córdoba, Córdoba, Spain; 4 Harvard T.H. Chan School of Public Health, Boston, MA 02115 USA; 5 Department of Medicine, Aging Institute, University of Pittsburgh School of Medicine, Pittsburgh, PA USA; 6 Department of Anesthesiology, University of Michigan, Ann Arbor, MI USA; 7 Molecular and Integrative Physiology, University of Michigan, Ann Arbor, MI USA; 8 Department of Physiology and Pharmacology, University of Toledo College of Medicine and Life Sciences, Toledo, OH USA

**Keywords:** Mitochondria, Metabolomics

## Abstract

The intrinsic aerobic capacity of an organism is thought to play a role in aging and longevity. Maximal respiratory rate capacity, a metabolic performance measure, is one of the best predictors of cardiovascular- and all-cause mortality. Rats selectively bred for high-(HCR) vs. low-(LCR) intrinsic running-endurance capacity have up to 31% longer lifespan. We found that positive changes in indices of mitochondrial health in cardiomyocytes (respiratory reserve, maximal respiratory capacity, resistance to mitochondrial permeability transition, autophagy/mitophagy, and higher lipids-over-glucose utilization) are uniformly associated with the extended longevity in HCR vs. LCR female rats. Cross-sectional heart metabolomics revealed pathways from lipid metabolism in the heart, which were significantly enriched by a select group of strain-dependent metabolites, consistent with enhanced lipids utilization by HCR cardiomyocytes. Heart–liver–serum metabolomics further revealed shunting of lipidic substrates between the liver and heart via serum during aging. Thus, mitochondrial health in cardiomyocytes is associated with extended longevity in rats with higher intrinsic exercise capacity and, probably, these findings can be translated to other populations as predictors of outcomes of health and survival.

## Introduction

The intrinsic aerobic exercise capacity of an organism can play a role in determining healthspan and lifespan, as suggested by the predictive power of low exercise capacity on premature morbidity and mortality for healthy adults, as well as those with cardiovascular disease^1^. Hill^2^ proposed that maximal respiratory rate capacity (VO_2_max) is the single best measure of cardiorespiratory performance and a strong and quantitatively reliable predictor of cardiometabolic health and all-cause mortality, and its clinical usefulness has withstood the test of time^3^ (see ref. ^4^ for review).

As a metric of maximal metabolic rate (MMR), VO_2_max describes the overall flow rate through the respiratory system, encompassing performance from the lung and circulation to muscle mitochondria. Mitochondrial respiration scales as a function of body size both at rest and maximal metabolic performance, and the aerobic capacity of locomotor muscle scales with MMR, which, in turn, scales with the surface area of inner mitochondrial membranes, where oxidative phosphorylation (OxPhos) takes place, underscoring the fundamental role of mitochondrial molecular mechanisms in determining aerobic capacity^5^.

A healthy mitochondrial network function might be of critical importance for limiting cellular damage accumulation, slowing the rate of senescence, and leading to improvements in healthspan and lifespan (reviewed in ref. ^6^). The susceptibility to mitochondrial permeability transition pore (mPTP) opening, a proxy of mitochondrial fitness, is a key player in the integration of mitochondrial energy metabolism to cell life and death decisions^7–9^.

Autophagy is one of the most important cytoplasmic recycling mechanisms in keeping cellular homeostasis by turning over impaired organelles (e.g., mitochondria) and degrading unwanted proteins (e.g., damaged or misfolded), lipids, and carbohydrates that, in turn, can provide additional energy^10–12^. Direct modulatory roles of autophagy in the aging process^13^ and in deceleration of biological clocks^12^ have been proposed. Autophagy of mitochondria, termed “mitophagy”^14^, occurs in the context of pronounced energy deficits, during developmentally regulated removal of these organelles in the course of differentiation of certain cell types, and regulates the natural turnover and quality control of mitochondria via selective targeting and removal of dysfunctional mitochondria or their fission fragments^15^.

Degradation of mitochondrial quality via reduced mitophagy, mitochondrial fusion–fission dynamics, and biogenesis, presumably results in the overall energetic-redox impairment of the mitochondrial network, as shown by mitochondrial populations isolated from organs affected by metabolic disease^16–18^ or by experimental ablation of fission proteins^19,20^. Mitochondrial quality control is altered by both nutrient excess and physical inactivity, with the liver^21^, cardiac, and skeletal muscle^22–24^ among the most affected. In the human body, the heart utilizes several-fold higher amounts of O_2_ on a specific basis (per gram of wet weight) than other organs such as skeletal muscle, brain and the lung^16,25^. Thus, the heart faces the highest degree of exposure to possible oxidative damage^16,26^. Mitochondria must serve as a reliable energy supply, by harnessing not only essential redox and phosphorylation energy but also must limit reactive oxygen species (ROS) within physiological limits compatible with signaling^27–31^. Poor eating habits^32^, metabolic syndrome^23,24,33^, and lack of physical activity^22,34^ account to a great extent for a high vulnerability of the heart to redox damage and high morbidity and mortality from cardiovascular complications.

Cardiomyocytes from aged hearts have a substantially lower threshold for ROS-induced ROS release and for opening of the mPTP^35^. Although a direct cause-effect relationship between mitochondrial dysfunction and disease is well established, their contributory role to quality of life and longevity is less well known (reviewed in ref. ^6^). Several lines of evidence converge on the idea that dysfunctional mitochondria accumulate as cells age, presumably due in part to the loss of appropriate mitophagy, promoting excess ROS that leads to spiraling molecular and organelle damage^36^.

The high- and low-capacity runners (HCR and LCR) were originated from a founder population of 168 genetically heterogeneous rats derived from outcrossing 8 inbred strains (N:NIH stock)^37^. The rats were selected by their performance in running treadmill tests under controlled acceleration. Originally, Koch and Britton^38^ hypothesized that artificial selection based on intrinsic aerobic capacity would yield divergent animal models in disease risks. After 28+ generations of selection, HCR and LCR diverged in blood pressure, body mass index, lung capacity, lipid and glucose metabolism, in addition to running capacity^39^. Reported evidence also suggested that OxPhos function and lipid metabolism segregate with running capacity and disease-risk phenotypes^38,40–42^.

The present work explores the potential impact of mitochondrial health on cardiorespiratory performance in two different rat strains characterized by differing intrinsic exercise capacity and lifespan. Specifically, we performed a cross-sectional study on aging in female HCR and LCR—young (6 months), middle-age (17 months), and old (24 months) rats, hypothesizing that mitochondrial health will be increased in the longer-lived HCR vs. LCR to sustain their higher aerobic capacity as a function of aging, for example, due to improved mitochondrial quality control and function because of better maintenance of autophagy/mitophagy mechanisms. The hypothesis was tested with parallel measurements in isolated cardiac myocytes of (i) mitochondrial respiratory reserve (Rres), (ii) maximal respiratory capacity (VO_2_max) in the presence of two different substrates (glucose and palmitate (Palm)) alone or in combination, (iii) autophagy/mitophagy, (iv) accumulation of intra-lysosomal debris, (v) mitochondrial stress resistance (i.e., fitness, as gauged by sensitivity to opening of the mPTP by ROS), and (vi) cross-sectional metabolomics. Metabolomics of the heart, liver, and serum were performed to evaluate the integrated, organism-level metabolic response, and the relative effect of aging in HCR and LCR.

## Results

### Rres in HCR and LCR as a function of substrate and age

**Fig. 1:**
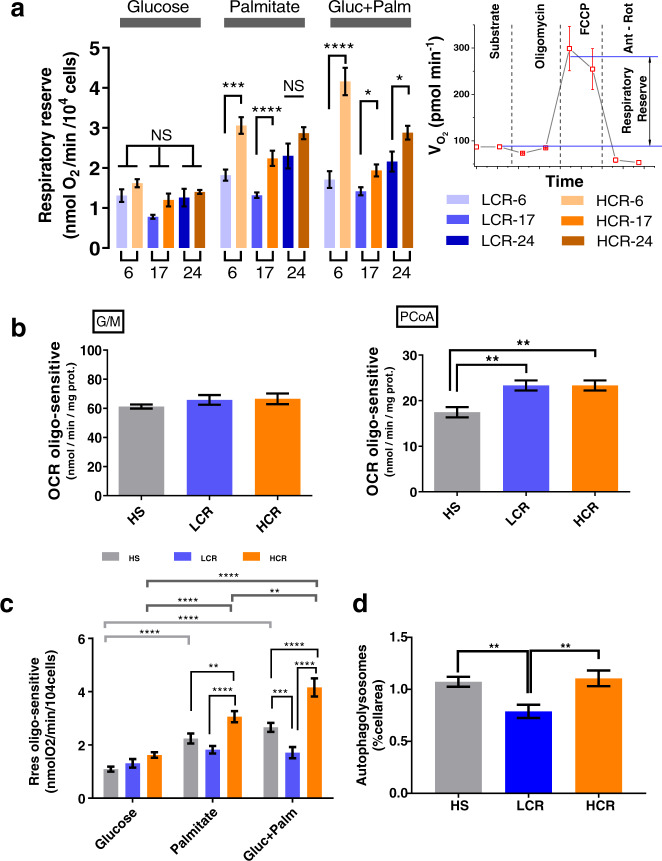
Respiratory reserve (Rres) in isolated cardiomyocytes from HCR and LCR as a function of substrate and age. a Rres, defined as the difference between the oxygen consumption rate (OCR) OCR_FCCP_ minus the OCR_substr_ (inset; see also Supplementary Fig. 1), was quantified in ventricular cardiomyocytes isolated from HCR or LCR rats at the indicated ages (6, 17, or 24 months old). Cardiomyocytes were assayed in the presence of 5 mM glucose or 0.2 mM palmitate bound to fatty acid-free bovine serum albumin, 4 : 1, or the combination of both substrates added in the first part of the assay (marked as substrate in the inset) before subsequent additions as described in “Cardiomyocytes isolation and high-throughput respiratory measurements” in “Methods”. See related Supplementary Fig. 1 for OCR measurements, full experimental design, experiments/*n*-values, and Supplementary Figs 2 and 3 for additional two-way ANOVA with Tukey’s multiple comparison test (GraphPad Prism 8.0) and all data points. **b** OCR-oligomycin sensitive obtained with mitochondria isolated from the hearts of 6-month-old HS (3 rats/experiments, *n* = 62), LCR (4 rats/experiments, *n* = 85), or HCR (4 rats/experiments, *n* = 90) rats, and assayed in the presence of glutamate/malate (G/M) or palmitoyl CoA (PCoA)/malate as detailed in “Methods”. See related Supplementary Fig. 5 including all data points. The statistical analysis corresponds to one-way ANOVA with Tukey’s multiple comparison test (GraphPad Prism 8.0). **c** Rres of cardiomyocytes isolated from 6-month-old HS (3 rats/experiments, *n* = 66–72), LCR (4 rats/experiments, *n* = 37–47), or HCR (4 rats/experiments, *n* = 37–48) rats, as indicated in the presence of glucose or palmitate or their combination (see “Methods” for details and related Supplementary Fig. 6). Two-way ANOVA analysis with Tukey’s multiple comparison test was performed with GraphPad Prism 8.0 using substrate and strain as factors. **d** Autophagy in cardiomyocytes isolated from 6-month-old HS (3 rats/experiments, *n* = 78), LCR (4 rats/experiments, *n* = 52), or HCR (4 rats/experiments, *n* = 53) rats (see “Methods” for details and related Supplementary Fig. 7a, which, in addition, shows measurements of *t*_m_PTP (Supplementary Fig. 7b) and lipofuscin (Supplementary Fig. 7c). The statistical analysis corresponds to one-way ANOVA with Tukey’s multiple comparison test (GraphPad Prism 8.0). In all cases, data are represented as mean ± SEM and the statistical significance is indicated by *p < 0.05; **p < 0.01; ***p < 0.001; ****p < 0.0001; NS not significant. FCCP Trifluoromethoxy carbonylcyanide phenylhydrazone.

Mitochondrial Rres—operationally defined as the maximal respiratory capacity after subtraction of baseline respiration—is a metric that can be used as an index of mitochondrial health^17^. We assessed Rres in isolated cardiac myocytes from LCR and HCR rats where it was quantified in the presence of the two major substrates that fuel heart function, glucose (Gluc) and, more abundantly, fatty acids (e.g., Palm), alone or combined, utilizing high-throughput measurements of O_2_ consumption rate (OCR) in cardiomyocytes. Figure 1a shows that in Gluc alone, Rres did not exhibit significant differences between LCR and HCR at any age studied. In contrast, HCR cardiomyocytes exhibit significantly higher Rres than LCR in the presence of Palm or Gluc + Palm, across the ages, except in Palm at 24 months where a nonsignificant trend was observed. Detailed normalized OCR measurements performed in the two strains at 6, 17, and 24 months of age in the presence of the different substrates are shown in Fig. 1, at each stage of the protocol schematized in the inset of Fig. 1a, and the overall experimental design that includes number of experiments and *n*-values (Supplementary Fig. 1h; see also Supplementary Fig. 4 with examples of raw, non-normalized OCR values across ages).

Both strains, LCR and HCR, exhibit changes in Rres as a function of age vs. substrate or age vs. strain (Supplementary Fig. 3). In Supplementary Fig. 3a, two-way analysis of variance (TWA) indicates that the influence of age is highly significant but only explains ~7% of the Rres changes when compared with either substrate (Supplementary Fig. 3a) or strain (Supplementary Fig. 3b). Moreover, the highly significant influence of the substrate is twofold larger in HCR vs. LCR (15% vs. ~7%, respectively) and shows a significant interaction with age in HCR but not in LCR (Supplementary Fig. 3a). TWA of cardiomyocytes maximal respiratory capacity (VO_2_max) (age vs. substrate for both strains), a metric closely related to Rres (see “Methods”), shows similar results (Supplementary Fig. 2); in HCR, the substrate factor plays a bigger role than age (~17% vs. ~3%), whereas in LCR the inverse is true, i.e., age is bigger than substrate (~12% vs. ~6%). Similar to Rres (Supplementary Fig. 3), HCR’s VO_2_max exhibits a significant interaction between age and substrate, but not in LCR (Supplementary Fig. 2).

High-throughput measurements of OCR in isolated heart mitochondria from the two strains, fueled with glutamate/malate (G/M) or palmitoyl CoA (PCoA) (Fig. 1b and Supplementary Fig. 5), partially recapitulated the results obtained in intact cardiomyocytes. Like in cardiomyocytes, substrates derived from glucose metabolism (G/M, Fig. 1b), elicited mitochondrial OCRs that were not significantly different between 6 months old LCR and HCR (Fig. 1, compare panels a and b). However, unlike cardiac myocytes (Fig. 1a), isolated heart mitochondria from both LCR and HCR hearts in the presence of PCoA sustained significantly higher OCR than in the heterogeneous outbred stock colony (HS) in the presence of PCoA (Fig. 1b).

The strain-dependent lipid utilization prompted us to test whether the association between high running capacity and substrate utilization was already present at young age. At 6 months of age, we assessed Rres in LCR and HCR strains and in the HS^37^ from which the aforementioned strains were derived^38^. Although the HS rats cannot serve as an appropriate Control group for the HCR/LCR experiments (because of the inherently diverse genetic background of HS), we provide HS data just as a contrasting animal model with respect to the metrics assessed in LCR and HCR at young age. Compared to Gluc alone, Rres increased significantly in cardiomyocytes of HCR but not LCR in the presence of Palm, and more so with Gluc + Palm (Fig. 1c). TWA revealed that both factors, strain, and substrate, share 16% and 11% of the influence over Rres, respectively (Supplementary Fig. 6).

Together, the data show that differences in metrics of cardiomyocytes aerobic capacity, Rres and VO_2_max, are significantly different if Palm is a substrate, in favor of HCR vs. LCR. As a factor, age plays a significant but relatively weaker role compared to substrate in those differences. The role of substrate in the differences between strains, particularly in the presence of the FA Palm alone or with glucose, is higher in HCR vs. LCR while age plays a bigger role in LCR vs. HCR. At young age, the influence of substrate and strain on Rres are already present and higher in HCR vs. LCR.

### Autophagy/mitophagy and mitochondrial fitness are better preserved in cardiomyocytes from HCR vs. LCR

Next, we investigated whether a higher Rres was associated with mitochondrial fitness and turnover linked to autophagy/mitophagy. These metrics were assessed in parallel with Rres and VO_2_max employing live-cell imaging and electron microscopy (EM) of cardiac myocytes from all age groups. In addition, accumulation of lipofuscin, a biomarker of molecular/organellar recycling impairment, was also measured.

**Fig. 2:**
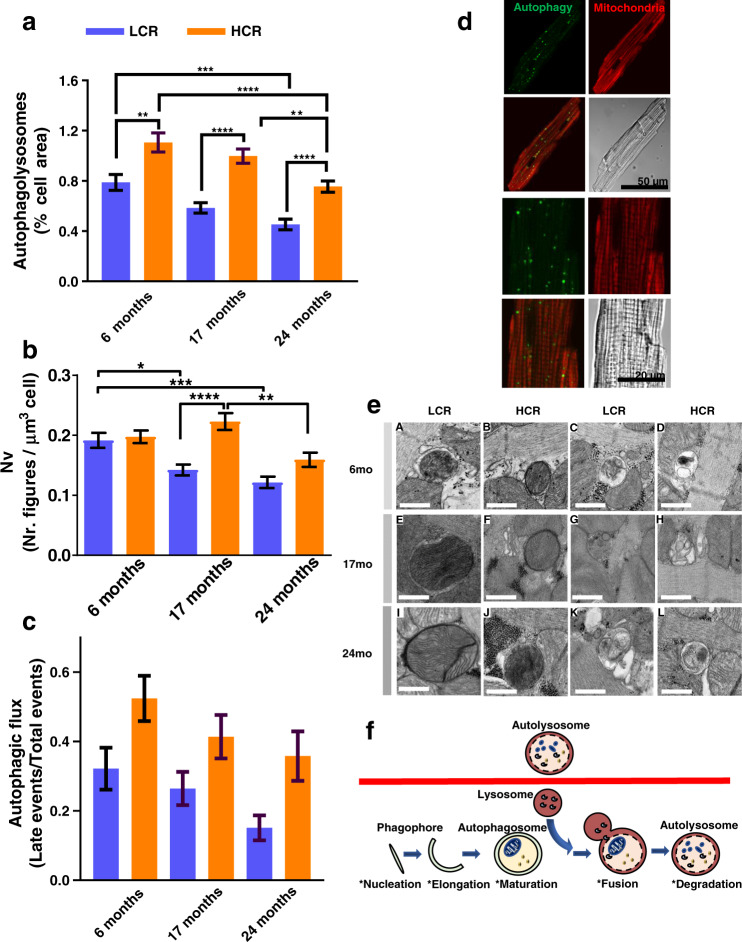
Confocal live fluorescence imaging and electron microscopy (EM) of autophagy/mitophagy in LCR and HCR cardiomyocytes during aging. a Depicted are the levels of autophagy/mitophagy quantified as % cell area covered by autophagy vesicles in isolated cardiomyocytes from 6 (LCR: 4 rats/experiments, *n* = 54; HCR: 4 rats/experiments, *n* = 53), 17 (LCR: 4 rats/experiments, *n* = 66; HCR: 5 rats/experiments, *n* = 70), and 24 (LCR: 5 rats/experiments, *n* = 94; HCR: 7 rats/experiments, *n* = 112) months old rats, using high-resolution confocal microscopy of formed autophagic vesicles labeled by the fluorescent CYTO-ID for autophagy detection (green) according to manufacturer’s instructions (see “Confocal fluorescence imaging” and “Electron microscopy” in “Methods”) and mitochondrial staining with 100 nM TMRM (red). See related Supplementary Fig. 8a for additional two-way ANOVA with Tukey’s multiple comparison test (GraphPad Prism 8.0) and all data points. **b** Quantification of total autophagy/mitophagy figures (autophagosomes [early] + autophagolysosomes [late]) observed by EM and normalized with respect to the cell volume using stereological procedures (see “Confocal fluorescence imaging” and “Electron microscopy” in “Methods”) in samples of LCR and HCR cardiomyocytes from 6 (LCR: 2 rats/experiments, *n* = 40; HCR: 2 rats/experiments, *n* = 40), 17 (LCR: 2 rats/experiments, *n* = 48; HCR: 2 rats/experiments, *n* = 47), and 24 (LCR: 2 rats/experiments, *n* = 37; HCR: rats/experiments, *n* = 35) months old rats. **c** Quantification of autophagic flux (late/(early + late)) figures observed by EM in samples of LCR and HCR cardiomyocytes from 6 (LCR: 2 rats/experiments, *n* = 38; HCR: 2 rats/experiments, *n* = 35; NS, *p* = 0.14), 17 (LCR: 2 rats/experiments, *n* = 49; HCR: 2 rats/experiments, *n* = 41; NS, *p* = 0.34), and 24 (LCR: 2 rats/experiments, *n* = 38; HCR: 2 rats/experiments, *n* = 27; NS, *p* = 0.17) months old rats. For **b**, **c**, see Supplementary Fig. 9a, b for additional two-way ANOVA with Tukey’s multiple comparison test (GraphPad Prism 8.0) and all data points. **d** Representative examples of cardiomyocytes; clockwise, from top left: CYTO-ID-stained autophagic vesicles, TMRM, transmission light microscopy, merge, and detailed (zoomed) image of a cardiomyocyte: CYTO-ID-stained autophagic vesicles, TMRM, transmission light microscopy, merge. **e** Representative electron micrographs of autophagy/mitophagy figures in cardiomyocytes isolated from LCR and HCR hearts at the indicated ages. Bar size = 0.5 µm in all panels. “Early” figures of autophagosomes (depicted in **A**, **B**, **E**, **F**, **I**, **J**) were identified by its content (morphologically intact, mostly mitochondria) and a double limiting membrane separated by a narrow electron-lucent cleft. “Late” figures of autophagolysosomes (depicted in **C**, **D**, **G**, **H**, **K**, **L**), delimited by single membranes, represent an advanced stage of autophagosomes where a lysosome has merged, and the content comprises partially degraded, irregular, electron-dense or pale material depending upon time of formation. **f** Interpretative scheme of autophagy/mitophagy describing the stages of this process measured by EM quantification. In all cases, data are represented as mean ± SEM. The statistical significance is indicated by *p < 0.05; **p < 0.01; ***p < 0.001; ****p < 0.0001; NS not significant.

Autophagy was quantified as accumulation of autophagic vesicles, visualized by combining vital-dye staining with confocal microscopy imaging of cardiac myocytes subsets from LCR and HCR in which Rres and VO_2_max were measured in parallel. At a young age, autophagy was already significantly higher in HCR vs. LCR (Fig. 1d and Supplementary Fig. 7a) and was resistant to oxidative stress-mediated mPTP opening (Supplementary Fig. 7b), whereas HS resembled HCR rather than LCR. The higher autophagy/mitophagy exhibited by HCR vs. LCR at 6 months (Figs 1d and 2a, c) might, in part, explain the difference in respiration in the presence of lipid, observed between intact cardiomyocytes (Fig. 1a), and isolated mitochondria (Fig. 1b), which in the latter would be substantially altered by the isolation procedure. Aspects of enhanced mitochondrial function in intact cardiomyocytes that could be lost by cell disruption include loss of interactions between mitochondria, responsible for their network behavior, or the deficit of requisite cytoplasmic factors and/or signaling^6,43^.

Cross-sectionally, strain and age explain 11% and ~8% of changes in autophagy, respectively (Fig. 2a and Supplementary 8a). Importantly, autophagy is significantly higher in HCR vs. LCR at all ages tested (Supplementary Fig. 8a). The results of live-cell imaging were consistent with those obtained with quantitative EM, where total ([early] autophagosomes + [late] autophagolysosomes) (Fig. 2e) and relative (late/[early + late]) were determined as metrics of autophagy/mitophagy (Fig. 2b) and autophagic flux (Fig. 2c), respectively, in a subset of the same pool of cardiac myocytes. The gallery of images displayed in Fig. 2e shows representative EM micrographs obtained from isolated cardiomyocytes depicting mitophagy figures, in the two first columns, at 6, 17, and 24 months of age in LCR: A, E, L and HCR: B, F, J, respectively, and of autophagy, in the third and fourth columns, at the indicated ages and strain. “Early” figures of autophagosomes are surrounded by a double limiting membrane and the content, which in A, B, E, F, I, J, correspond to morphologically intact mitochondria. “Late” figures of autophagolysosomes (illustrated in C, D, G, H, K, L), surrounded by a single membrane, represent an advanced stage where the autophagosome has merged with a lysosome and its content becomes partially degraded and irregular, electron dense, or pale, depending on the time of formation.

The results show that, at young age, the autophagic flux exhibited a nonsignificant higher trend in HCR vs. LCR (Fig. 2c), in agreement with live-cell imaging (Fig. 2a), although the total number of autophagy/mitophagy figures from both strains was similar (Fig. 2b); the latter can be explained by the fact that the CytoID probe labels autophagolysosomes, whereas EM counts mitophagy figures, in addition to autophagosomes and autophagolysosomes (Fig. 2e, f). The trend of increased autophagic flux in HCR vs. LCR was maintained across ages, accompanied by a significantly 36% higher total autophagy/mitophagy figures per constant volume density (data not shown) at mid-age, and a nonsignificant trend at the oldest age (Fig. 2b).

We also investigated the potential role of mitochondrial biogenesis using EM micrographs to determine mitochondrial numbers, size (area), and fractional area occupied by mitochondria^44^. Only at 17 months of age, HCR exhibited a significantly higher 22% number (Supplementary Fig. 9c) of smaller mitochondria (Supplementary Fig. 9e) that covered a similar cell area (Supplementary Fig. 9d) compared to LCR; however, these metrics were unchanged at other ages. As at 17 months Rres/VO_2_max (Fig. 1a and Supplementary Fig. 2a) and autophagy/mitophagy (Fig. 2) were higher 41/45% and 42% (confocal)/33% (EM) in HCR vs. LCR, respectively, we conclude that, except at middle age, where both autophagy/mitophagy and mitochondrial biogenesis contribute to the higher respiration observed, turnover rather than biogenesis was the main driver of mitochondrial quality in HCR. TWA further showed that age and strain exert ~7% and ~1%, respectively, influence on the variance of mitochondrial number (Supplementary Fig. 9c).

**Fig. 3:**
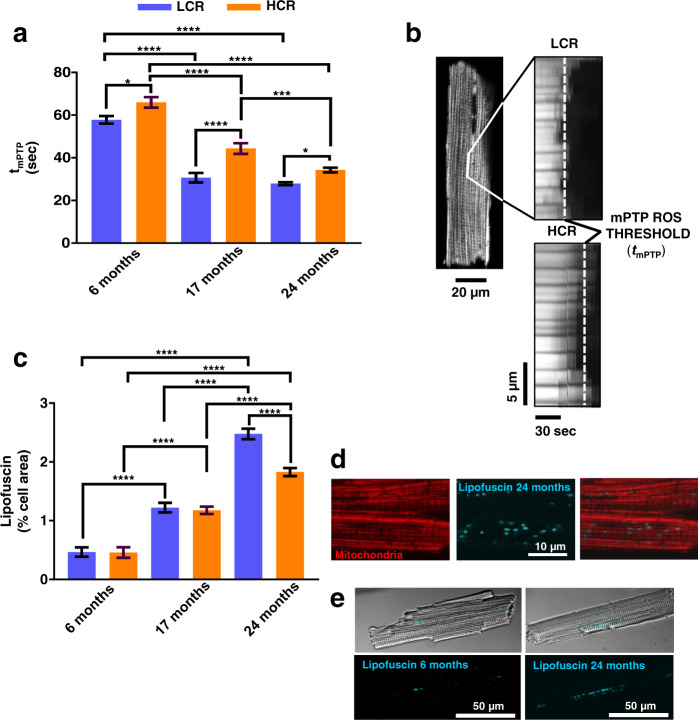
Confocal live fluorescence imaging of mitochondrial fitness and lipofuscin in LCR and HCR cardiomyocytes during aging. a mPTP-ROS threshold (*t*_mPTP_) as index of mitochondrial fitness was quantified in isolated TMRM-loaded cardiomyocytes from 6 (LCR: 4 rats/experiments, *n* = 47; HCR: 4 rats/experiments, *n* = 46), 17- (LCR: 4 rats/experiments, *n* = 44; HCR: 5 rats/experiments, *n* = 44), and 24 (LCR: 5 rats/experiments, *n* = 79; HCR: 7 rats/experiments, *n* = 91) months old rats, using high-resolution confocal fluorescent microscopy. *t*_mPTP_ reflects the average time required for the photoproduced ROS to cause mPTP opening induction. **b** Representative example of *t*_mPTP_ determination in LCR vs. HCR cardiomyocytes. **c** Lipofuscin quantification as % cell area covered by progressive age-dependent accumulation of lipofuscin as revealed by autofluorescence of cardiomyocytes from 6 (LCR: 3 rats/experiments, *n* = 55; HCR: rats/experiments, *n* = 56), 17 (LCR: 2 rats/experiments, *n* = 33; HCR: 2 rats/experiments, *n* = 34), and 24 (LCR: 5 rats/experiments, *n* = 80; HCR: 5 rats/experiments, *n* = 97) months old rats. **d** Zoomed image showing, in detail, accumulated lipofuscin as visualized by autofluorescence (blue) and TMRM-loaded mitochondria (red), and **e** representative examples of cardiomyocytes, full-scale, at 6 and 24 months of age. In all cases, data are represented as mean ± SEM. The statistical significance is indicated by *p < 0.05; **p < 0.01; ***p < 0.001; ****p < 0.0001; NS not significant.

To further explore the interplay of mitochondrial health and fitness, we determined the resistance to mitochondrial permeability transition, measured as the time (*t*, in seconds) to reach the ROS-induced ROS release threshold for mPTP opening (*t*_mPTP_). Indeed, *t*_mPTP_ was significantly longer in cardiomyocytes from age-matched HCR vs. LCR (Fig. 3a, b). TWA showed that age had a major impact (~50%) with strain exerting a relatively weak (~6%), but highly significant influence (Fig. 3a, b and Supplementary Fig. 8b). The resistance to opening of the mPTP with oxidative stress was significantly higher in HCR vs. LCR (Supplementary Fig. 8b), a result in agreement with better autophagy/mitophagy (Figs 2 and 3, and Supplementary Fig. 8a, b).

Accumulation of lipofuscin, an undegradable cellular “wear-and-tear” byproduct, increased dramatically with age in both strains but was significantly higher only in old (24 months) LCR vs. HCR (Fig. 3c, d and Supplementary Fig. 8c). In older animals, typically perinuclear lipofuscin granules exhibited a widespread cellular localization, whereas at 6 months of age lipofuscin accumulation in cardiac myocytes from both strains was comparable and negligible, appearing restricted to small perinuclear areas (Fig. 3e). TWA showed that age has a major impact (~55%) on lipofuscin accumulation, with strain exerting a negligible (~1%) effect (Fig. 3c and Supplementary Fig. 8c). The presence of lipofuscin granules in cardiomyocytes was independently confirmed by EM (data not shown).

Consistent with our initial hypothesis, autophagy/mitophagy and turnover are enhanced in cardiomyocytes of HCR vs. LCR, even from a young age (Fig. 1d), which underlies, at least in part, the significant differences in mitochondrial Rres, VO_2_max and fitness observed between both strains. Although there is an age-related decline in mitochondrial Rres and VO_2_max independent of rat strain, the relative enhancement of these metrics in HCR vs. LCR is sustained during aging. Furthermore, HCR’s Rres is positively enhanced by the influence of substrate (namely the combination of glucose with Palm) (Supplementary Fig. 3b) and by its interaction with age (Supplementary Fig. 3a, b). The ensemble of data indicates that, comparatively, mitochondrial turnover via autophagy/mitophagy (Fig. 2a–c and Supplementary Fig. 8a) rather than biogenesis (Supplementary Fig. 9c, d) appears to be the main driver of the mitochondrial health benefits in HCR vs. LCR (Supplementary Fig. 9c). A stronger influence of age vs. strain on the variance of *t*_m_PTP (50% vs. 6%) and lipofuscin (55% vs. ~1%) indicates that, together, these metrics behave as biomarkers of mitochondria and heart aging.

### Cross-sectional metabolomics of the heart–liver–serum from HCR vs. LCR across the age span analyzed

Our findings in cardiac myocytes led us to explore whether, in an organismal context, these insights were consistent with the heart’s metabolic status and the liver’s role to supply substrates for delivery by the circulation to the rest of the body. Utilizing metabolomics of the heart, liver, and serum, we specifically looked for a cardiac pattern of lipids utilization and their associated pathways/metabolites, as our cardiomyocyte data predicted. At the organismal level, we sought to see whether a meta-pattern of expected substrates delivered by the liver via the circulation overlaps with the pattern of substrate utilization by the heart.

**Fig. 4:**
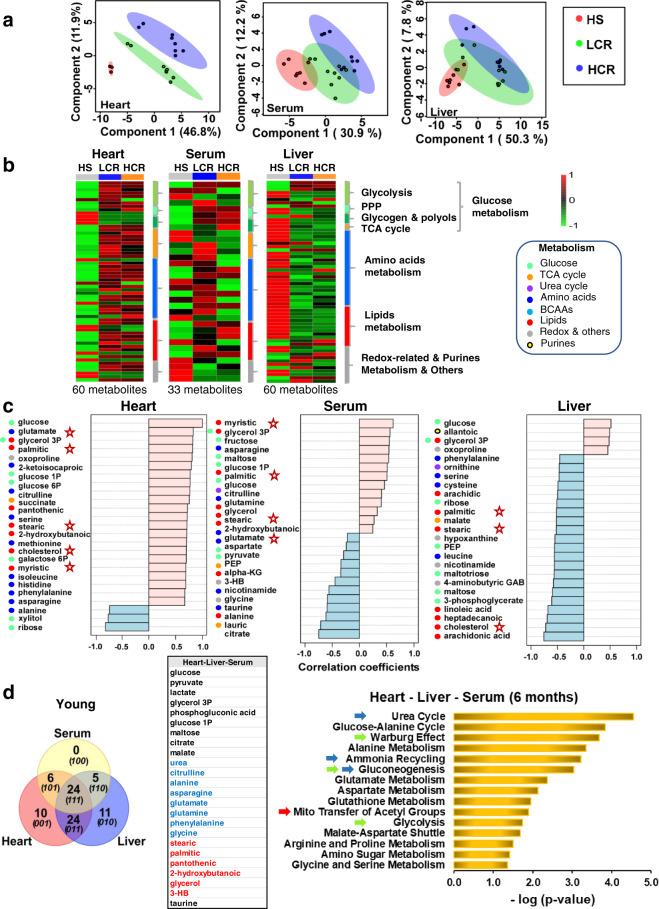
Systems metabolomics of heart, serum, and liver in 6 months age HS, LCR, and HCR rats. Untargeted metabolomics performed in young (6 months) age rat: heart (HS, *n* = 3; HCR, *n* = 8; LCR, *n* = 8), liver (HS, *n* = 8; HCR, *n* = 9; LCR, *n* = 9), and serum (HS, *n* = 7; HCR, *n* = 9; LCR, *n* = 9) samples. **a** Partial least-square discriminant analysis (PLS-DA) of significantly changed metabolites from the heart (left), serum (mid), and liver (right). **b** Average heatmaps of significantly changed metabolites from the heart (left), serum (middle), and liver (right) of HS (left lane), LCR (middle lane), and HCR (right lane) displayed as accumulation (red) or depletion (green) according to the pseudocolor scale on the right of the maps. The brackets and legend on the right of the heatmaps indicate the main metabolic pathways to which the metabolites belong. See also related Supplementary Fig. 10. **c** Correlation pattern plots highlighting the 25 most significantly changing metabolites for sequentially arranged HS-LCR-HCR meaning that the accumulation/depletion patterns correspond to HCR. Metabolite groups are denoted by different colors. Stars denote metabolites that are shunted between liver and heart according to the patterns of depletion/accumulation. **d** Venn diagrams of unique and shared metabolites between the heart, liver, and serum from HS-LCR-HCR at young (6 months) age (left panels) and bar graph of significantly enriched pathways (*p* < 0.05; right panel) by the “core” metabolome constituted by 24 shared metabolites, independently from organ and strain (middle table). For analysis details, see “Bioinformatic and statistical analyses” in “Methods”.

To test these ideas in the absence of aging effects, we first analyzed the heart–liver–serum metabolomics from LCR, HCR, and HS at a young (6 months) age. Using untargeted metabolomics, 60 (liver and heart) and 33 (serum) metabolites out of 136 that were detected/identified differed significantly between young HS, LCR, and HCR, as determined by a combination of univariate and multivariate statistics, including pairwise comparisons, TWA, partial least-square discriminant analysis (PLS-DA), heatmaps, and correlation patterns search (see “Methods” for details). In the heart, PLS-DA revealed substantial separation among the three strains, whereas in the serum and liver partial overlap was present among the three groups (Fig. 4a). Figure 4b and Supplementary Fig. 10 present the average level of significantly changed metabolites as heatmaps of the three strains in the three organs. The lipid metabolome revealed that the young HCR heart exhibits a pattern of metabolites compatible with lipids utilization, as judged from the analysis sequence, “HS-LCR–HCR,” which shows the correlation pattern of abundance/depletion of metabolites (Fig. 4c) corresponding to HCR. The HCR heart is enriched with palmitic, stearic, myristic, cholesterol, and pantothenic, the latter a precursor of CoA biosynthesis essential for β-oxidation, and the continuous function of the tricarboxylic acid (TCA) cycle. Oppositely, the liver becomes depleted of palmitic, stearic, arachidic, linoleic, heptadecanoic, cholesterol, and arachidonic acid, as serum is concomitantly enriched in palmitic, stearic, glycerol and depletion of lauric and the ketone body 3-hydroxybutyrate (3-HB) (Fig. 4c, marked by stars). Consequently, the heart’s lipids metabolome pattern is part of a meta-pattern given by the consistent enrichment/depletion changes in metabolites from liver and serum, as revealed by correlation patterns (Fig. 4c).

With respect to amino acids, the liver becomes depleted of phenylalanine, serine, cysteine, and leucine, whereas glutamate, serine, methionine, isoleucine, histidine, phenylalanine, and asparagine become abundant in the heart, where the accumulation of citrulline is consistent with activation of the Urea Cycle, suggesting amino acids’ anaplerotic feeding of the TCA cycle (Fig. 4d). The enrichment of glucose in the heart, liver, and serum (Fig. 4c) is consistent with shunting of this substrate from liver to heart as expected from the higher energy demand and mitochondrial energetic performance in the HCR (Fig. 4b, middle panel, and c).

Venn diagram analysis unveiled a core group of 24 shared, significantly changed (*p* < 0.05; false discovery rate (FDR) < 0.05), metabolites, i.e., independently from organ (heart–liver–serum) and strain (HS-LCR-HCR), which was subjected to pathways enrichment analysis (Fig. 4d, right panel). The results show that, except for gluconeogenesis and glucose-alanine cycle that happen only in the liver, amino acids metabolism involving urea cycle, ammonia recycling, anaplerosis, and lipid metabolism implicating transfer of acetyl groups into mitochondria and fatty acids degradation, all are significant (*p* < 0.05) and pathways common to the heart and liver (Fig. 4d, right panel).

The coherent pattern of fuel metabolites’ (glucose, lipids, and amino acids) abundance/depletion and pathways at the “core” of the interaction of heart–liver–serum in young rats are consistent with a meta-pattern of coordinated metabolic response between the heart and the liver, independently from strain and organ.

**Fig. 5:**
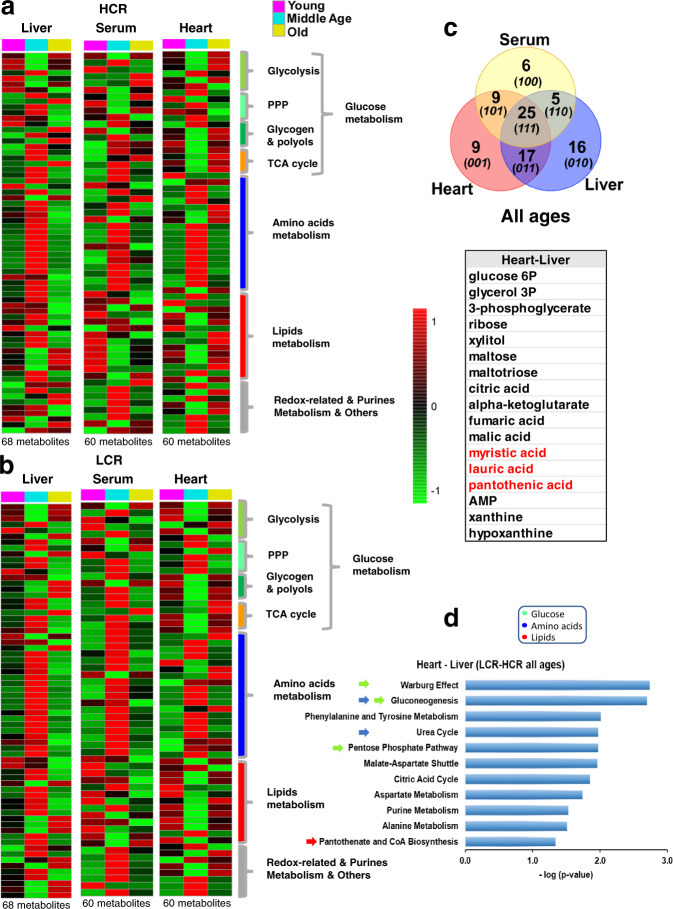
Cross-sectional heatmaps of significantly changed metabolites from heart, serum, and liver in HCR and LCR rats as a function of age. Average heatmaps of significantly changed metabolites from HCR (**a**) and LCR (**b**) in the heart at young, 6 months (HCR, *n* = 8; LCR, *n* = 8), middle, 17 months (HCR, *n* = 7; LCR, *n* = 8), and old, 24 months (HCR, *n* = 6; LCR, *n* = 6) age; the liver at young, 6 months (HCR, *n* = 9; LCR, *n* = 9), middle, 17 months (HCR, *n* = 10; LCR, *n* = 11), and old, 24 months (HCR, *n* = 9; LCR, *n* = 8) age; and serum at young, 6 months (HCR, *n* = 9; LCR, *n* = 9), middle, 17 months (HCR, *n* = 8; LCR, *n* = 8), and old, 24 months (HCR, *n* = 9; LCR, *n* = 8) age, ordered according to metabolic pathways. The pseudocolor scale (1 to −1 on the right of the heatmaps) reflects accumulation (red) or depletion (green) for each tissue at each age. See also related Supplementary Figs 11 and 12. **c** Venn diagrams of the unique and shared significant metabolites from serum and heart-liver of LCR-HCR across ages. The table (bottom) shows the 17 metabolites shared by the heart and liver, independently from strain and age, which were subjected to pathways enrichment analysis. **d** Bar graph of enriched pathways (*p* < 0.05) by significant metabolites shared by heart and liver. For analysis details, see “Bioinformatic and statistical analyses” in “Methods”.

Next, we explored whether aging differentially affects metabolism in HCR vs. LCR and whether specific metabolic changes found in cardiomyocytes/mitochondria reflect remodeling of heart metabolism. To this goal, we utilized a comparative systems metabolomics approach of the heart, liver, and serum from HCR and LCR across the age span studied. Figure 5a, b depicts the heatmaps of significantly changed metabolites compared for pathways, in three organs of the two strains as a function of age. Out of 136 metabolites detected/identified, 60 (heart and serum) and 68 (liver) were significantly different as a function of age (Fig. 5a, b and Supplementary Figs 11 and 12). Seeking which metabolic pathways are shared between the heart–liver across the lifespan of LCR/HCR, we applied Venn diagrams of the significant metabolomes (FDR < 0.05; *p* < 0.05) from the serum and heart–liver of LCR-HCR across ages (Fig. 5c). We found that 17 metabolites were shared between heart–liver, independently from strain and age, which when subjected to pathways enrichment analysis revealed significantly changed (*p* < 0.05) the major macronutrients’ metabolism such as glucose (pentose phosphate, TCA cycle), amino acids (urea cycle, TCA cycle anaplerosis, gluconeogenesis, the latter in the liver only), and lipids (pantothenate and CoA biosynthesis) (Fig. 5d).

**Fig. 6:**
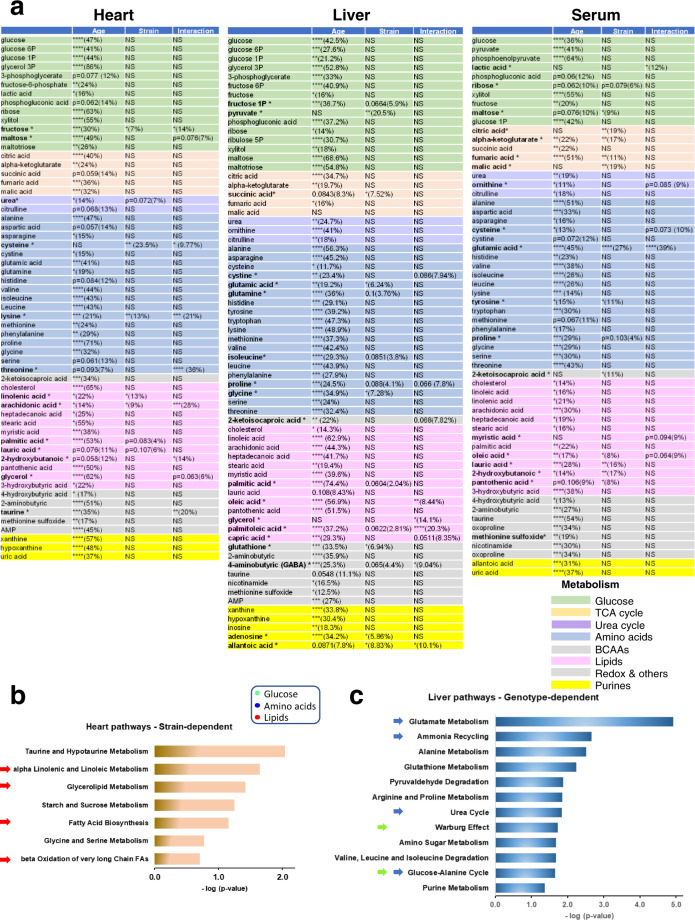
Comparative two-way ANOVA analysis of significantly changed metabolites in the heart–liver–serum of HCR and LCR, and the % of their variance influenced by age, strain, or their interaction. a Using two-way ANOVA analysis of significantly changed metabolites as a function of age, we determined the relative role of age vs. strain in all three organs. The relative significance of the influence exerted by age (young, middle, and old), genotype (HCR and LCR), and their interaction was determined in the heart, liver, and serum (top, left, middle and right tables, respectively). The color shading indicates the metabolic pathways to which metabolites belong, according to the legend at the bottom right. As most metabolic intermediates show variations with age, an #, next to the metabolite name, has been added to highlight the metabolites displaying significant differences due to the strain or the interaction between age and strain. Two-way ANOVA analysis with Tukey’s multiple comparison test was performed with GraphPad Prism 8.0 using age and strain as factors. In all cases, the statistical significance is indicated by *p < 0.05; **p < 0.01; ***p < 0.001; ****p < 0.0001, and the percentage of the variance due to the factor (age or strain) is expressed between brackets. Also, if 0.1 > *p* > 0.05, the *p*-value is informed together with the percent of variance. Number of experiments and *n*-values are informed in the legend of Fig. 5. **b**, **c** Bar graphs of significantly enriched pathways (*p* < 0.05) by strain-dependent metabolites in the heart (**b**) and liver (**c**). Pathways enrichment analysis was performed with the module “Pathways Analysis” of MetaboAnalyst 4.0^77^, a web-based resource for metabolomics analysis (see “Bioinformatic and statistical analyses” in “Methods”).

To dissect the effects of the strain from the age factor in the variance of each significantly changed metabolite, we performed TWA and found that it was, to a large extent, accounted for by age, except for a select group of metabolites that were influenced by strain and strain–age interaction within the heart and liver (Fig. 6a). Notably, among this selective group of metabolites, the heart exhibited a wider variety of lipids (linolenic, arachidonic, palmitic, lauric, arachidic, glycerol, and 2-hydroxybutanoic) followed by serum (myristic, oleic, lauric, 2-hydroxybutanoic, and pantothenic) and liver (oleic, glycerol, palmitoleic, and capric) (Fig. 6a). Separate pathways analysis of strain-dependent metabolites from each organ, revealed a predominant pattern of lipid metabolism-related (β-oxidation, fatty acid synthesis, ω-long-chain unsaturated linolenic, and linoleic) and associated pathways (redox-related taurine-hypotaurine and glycine-serine) in the heart (Fig. 6b), whereas liver metabolites aligned with expected functions (substrate supply, detoxification, and maintenance)-related pathways, e.g., amino acids, urea cycle, ammonia recycling, glutathione, and purines (Fig. 6c).

Finally, we sought to tease apart which of these pathways predominated in HCR vs. LCR. In an overview, middle-aged rats exhibited a clearly distinct metabolome pattern of abundance/depletion in the two organs and serum, leading us to focus on young and old (Fig. 5a, b and Supplementary Figs 11 and 12), where the metabolome and pathway pattern differences HCR vs. LCR appear to be quantitative rather than qualitative (Fig. 5a, b). Consequently, we employed a combination of correlation patterns of abundance/depletion of metabolites with average heatmaps.

**Fig. 7:**
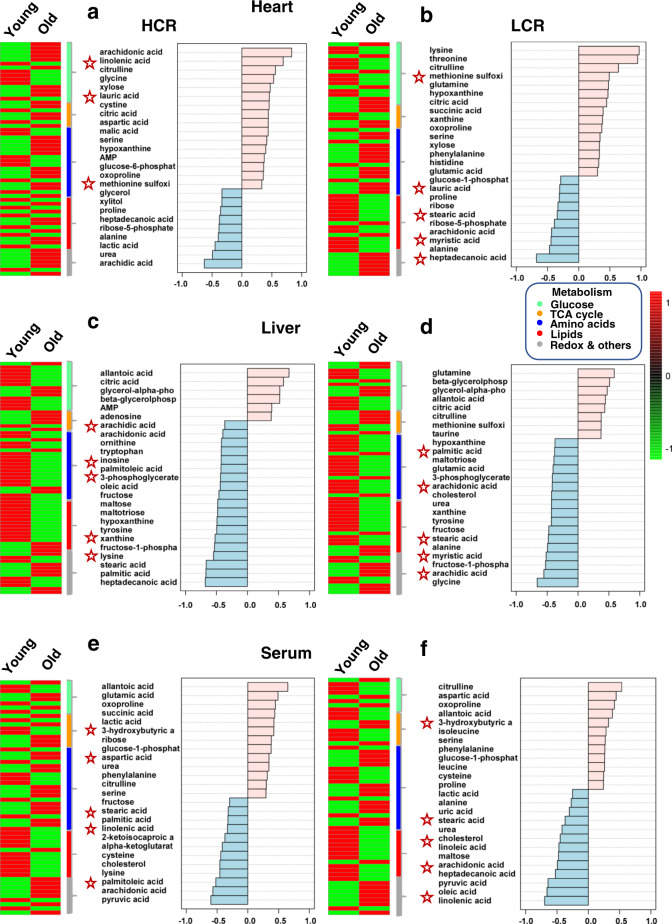
Comparative metabolomics of HCR vs. LCR, young vs. old, in the heart, liver and serum. Displayed are the average heatmaps and correlation plots of significantly changed metabolites in **a**, **b** the heart, **c**, **d** liver, and **e**, **f** serum from HCR (**a**, **c**, **e**) and LCR (**b**, **d**, **f**). In the correlation plots, the 25 most significantly changed metabolites (positively, in pink, or negatively, in light blue) are shown, according to the transition sequence young-old, meaning which metabolites tend to increase or decrease with old age in LCR or HCR in the respective organ. Stars highlight metabolites of significance mentioned in the main text. *n*-values corresponding to the different strains and organs at young and old age are informed in the legend of Fig. 5. See also related Supplementary Figs 13–15.

Figure 7 displays the analyses of HCR (Fig. 7a, c, e) and LCR (Fig. 7b, d, f) for each organ and serum. Age correlations of metabolites are shown in Fig. 7, right panels. In the HCR heart, lipids, notably polyunsaturated fatty acids (PUFAs; arachidonic, linolenic), were strongly and positively correlated with age (Fig. 7a, right panel). In contrast, in the LCR heart, more fatty acids such as lauric, stearic, arachidonic, myristic, and heptadecanoic were negatively correlated with age (Fig. 7b, right panel). As depicted by the heatmaps (Fig. 7a, b, left panels and Supplementary Fig. 13), a higher relative abundance of the ketone body 3-HB in the old HCR vs. LCR is consistent with higher lipid utilization by HCR runners (Fig. 7a, b). Although the hearts of HCR and LCR appear to be utilizing amino acids (as suggested by abundance patterns (Fig. 7a, b)), likely via anaplerosis, these routes are not significant as revealed by pathways analysis (Fig. 6b). Interestingly, branched-chain amino acids (BCAAs; valine, isoleucine, and leucine) were relatively depleted in the hearts of old vs. young HCR (Fig. 7a).

In the liver, the stark contrast between the HCR vs. LCR metabolomes with aging is mirrored by the inverse metabolite patterns in liver vs. heart (Fig. 7a–d, compare heart with liver, Supplementary Figs 13 and 14). Notably, age-associated metabolite depletion in the liver is accompanied by a concurrent heart enrichment in many of these metabolites (Fig. 7c, d). For example, in HCR liver (Fig. 7c, right panel), the negative correlation of non-esterified fatty acids (NEFAs) arachidic, arachidonic, stearic, palmitic, and heptadecanoic with age is concomitant with a positive correlation with the age of NEFA metabolites such as arachidonic, linoleic, and lauric in the HCR heart (Fig. 7a, c, compare the heart with liver, and Supplementary Figs 13 and 14). In contrast, in LCR (Fig. 7d, right panel), the depletion of NEFAs as a function of age (palmitic, arachidonic, cholesterol, stearic, myristic, arachidic) is not accompanied by concurrent cardiac enrichment, but rather by further depletion (lauric, stearic, arachidonic, myristic, heptadecanoic) (Fig. 7b, d, compare heart with liver, and Supplementary Figs 13 and 14).

In serum, both strains manifest positive age correlations of the ketone body 3-HB and concurrent negative age correlation of NEFAs (stearic, palmitic, and arachidonic), which appear enriched in HCR but not in LCR hearts (Fig. 7e, f and Supplementary Fig. 15).

Together, the ensemble of heart metabolomics data is consistent with a major finding of this study, i.e., that the high- vs. low-running capacity rats possess higher aerobic capacity sustained by significantly enhanced autophagy/mitophagy and turnover, mitochondrial fitness, and lipids utilization. The metabolomics data shows that although age exerts a strong influence on the heart and liver metabolomes, a select group of strain-dependent metabolites specifically enriches pathways from lipid metabolism, consistent with the cellular data. The enhanced lipids utilization exhibited by HCR cardiomyocytes agrees with the pattern of lipid metabolic pathways expressed in the HCR heart, and, moreover, with the meta-pattern associated with shunting of lipid substrates between liver and heart via serum at young and old age.

## Discussion

In the present study of high- vs. low-capacity female rat runners (HCR vs. LCR) differing by intrinsic exercise capacity and lifespan, we explored the links between mitochondrial health and the cardiorespiratory performance. We hypothesized that mitochondrial health will be improved in the longer-lived HCR vs. LCR to sustain their higher aerobic capacity as a function of aging due, at least in part, to a relatively enhanced autophagy/mitophagy. This hypothesis was tested using isolated cardiomyocytes and heart tissue in a cross-sectional study as a function of age, substrate, and strain. Main findings were that the HCR vs. LCR rats (i) possess enhanced mitochondrial health resulting in higher aerobic capacity as revealed by Rres and VO_2_max (Fig. 1 and Supplementary 1–6); and (ii) these augmented capacities were enabled by better (a) autophagy/mitophagy and turnover (Fig. 2a–c, and Supplementary Figs 8a and 9a, b) rather than by mitochondrial biogenesis (Supplementary Fig. 9c–e), (b) resistance to mPTP opening under oxidative stress, and (c) lipid utilization alone or in combination with glucose (Figs 1–3 and Supplementary Figs 7 and 8); (iii*)* for the differences between HCR vs. LCR in Rres and VO_2_max, substrate (15–17%) played a significantly bigger role than age (~7%) (Supplementary Figs 1 and 2), whereas in autophagy, strain and age were responsible for 11% and ~8%, respectively (Supplementary Fig. 8a); (iv*)* the influence of age was prominent in mitochondrial fitness (*t*_m_PTP, 50%) (Fig. 3a, b and Supplementary 8b), lipofuscin accumulation (56%) (Fig. 3c–e and Supplementary 8c), and metabolites (~20–55%) (Fig. 6a); (v) a coherent pattern of metabolites abundance/depletion and their corresponding pathways was found at the “core” of the interaction heart–liver–serum in young rats (i.e., independent from organ and strain), revealing a consistent pathways’ meta-pattern and substrates’ shunting between heart and liver (Figs 4 and 5); (vi) pathways from lipid metabolism in the heart (PUFAs, glycerolipids, FAs biosynthesis, and β-oxidation) are significantly enriched by a select group of strain-dependent metabolites (Fig. 6b), consistent with enhanced lipids utilization by HCR cardiomyocytes (Fig. 1) and the meta-pattern of lipidic substrates shunting between the liver and heart during aging (Figs 6 and 7).

The salutary preservation of mitochondrial health in HCR during aging could be an important underlying mechanism by which the numerous clinically relevant conditions (beyond running capacity and lifespan) that after 28 generations of selection^42^ characterized the divergence between LCR and HCR. Particularly, the improvement or preservation of cardiac and hepatic function^45,46^, stress-related behavioral strategies^47–49^, response to effects of diet^50,51^, and organ metabolism^46,52^. Germane to the present work, physical activity, energy expenditure, and lean body mass were all well preserved with age in HCR, whereas left ventricular function at myocardial and cardiomyocyte levels, Ca^2+^ dynamics, and mean blood pressure appeared more compromised in the LCR during aging^1^.

It is known that defective autophagy may favor the accumulation of lipofuscin and lipid droplets, which in turn can inhibit autophagy^53^. Significantly reduced accumulation of lipofuscin in old HCR vs. LCR indicated that better clearance of damaged molecules and/or organelles via autophagy occurs concurrently with improved mitochondrial Rres in HCR vs. LCR.

During short-term exercise, skeletal muscle of HCR continued ATP generation through OxPhos long past the point in which LCR reached maximal oxidative capacity^41^. This observation—where glycogen mobilization is delayed, while in the LCR glycogen contributes significantly to carbohydrate utilization without a comparable delay, which, in turn, causes its earlier depletion^41^—would be consistent with enhanced lipid utilization in HCR (Fig. 1a and Supplementary Fig. 2a).

Although our hypothesis was originally tested in cardiac myocytes, the insights obtained naturally led us to explore whether these agreed with the heart’s metabolism and the liver’s role in supplying substrates via the circulation. Metabolomics data showed that young rats possess a “core” group of significant metabolites (independent from organ and strain) together with major catabolic pathways of macronutrients (glucose, amino acids, and lipids), with most of them being shared by heart and liver (Fig. 4d). The pattern of metabolites abundance/depletion, that includes supply/utilization of lipids by the liver/heart, respectively, is part of a meta-pattern given by the consistent enrichment/depletion changes in metabolites from the liver and serum (Fig. 4c).

Aging was a major factor in metabolome changes (Fig. 6a), but we found that for heart and liver there is a specifically select group of metabolites that are influenced by strain and/or strain–age interaction. Remarkably, the heart exhibited a distinct (from the liver), predominant pattern of pathways of lipids metabolism, e.g., β-oxidation, ω-PUFAs, FA synthesis, and redox-related, e.g., taurine-hypotaurine (Fig. 6b, c). Further analytical dissection unveiled that during aging HCR vs. LCR show a cardiac pattern of lipidic metabolites abundance/depletion coherent with a meta-pattern of lipid substrates’ supply from liver to heart. Together, the ensemble of metabolomics data and cardiac myocytes respiration measurements support our conclusion that lipids, beyond Palm, are the major fuels of mitochondrial respiration in HCR vs. LCR heart, either alone or synergistically with glucose (Fig. 1a and Supplementary 2a), and consistent with these substrates’ coordinate shunting between the liver and heart (Figs 4 and 5).

As it has been shown that aberrant metabolism of BCAAs contributes to insulin resistance in humans^54,55^, the relative depletion of BCAAs in the old vs. young HCR heart reveals a potentially salutary trait (Fig. 7). There is a tightly linked utilization of fatty acids and BCAAs, which in the heart, as well as the skeletal muscle, is important because both are avid consumers of circulating fatty acids. When faced with reduced supply of lipids, these organs likely maintain TCA cycle flux by increasing oxidation of BCAAs, as has been revealed by the acute effect of increased BCAA oxidation caused by insulin; the latter is likely due to the inhibitory effect of lipolysis in white adipose tissue, which suppresses mobilization of fatty acids for consumption by other organs^56^.

The metabolomics data also suggest a possible beneficial association between the anti-inflammatory role of PUFAs^57,58^ and their significant abundance in the old heart of HCR vs. LCR. Together, the facts that: (i) the long-lived HCR exhibits higher autophagy/mitophagy and (ii) autophagy can mitigate inflammatory reactions through macrophage-mediated clearing of apoptotic cells, maintenance of intracellular ATP levels required for signaling pathways leading to tagging and disposal of dying cells, elimination of dysfunctional mitochondria, inhibition of proinflammatory signaling, and the activation of the NLRP3 inflammasome^36,59^, are consistent with the possible anti-inflammatory action, in addition to energy supply, of abundant PUFAs arachidonic and linolenic in the heart of old HCR.

Lipid homeostasis plays a fundamental role in autophagy regulation, both at the level of lipid signaling-mediated regulation through transcriptional factors and membrane lipid composition^60^. As a matter of fact, supplementation with ω-6 PUFAs extends worm lifespan by activating autophagy^61^, a mechanism that might be evolutionarily conserved, because ω-6 and other PUFAs induce autophagy in human cell lines^61,62^. Notably, several lines of evidence indicate that reproduction, resistance to starvation stress, and lifespan might be energetically linked through lipid metabolism^63^. In this context, autophagy engages lipid metabolism as a mechanism to extend lifespan in response to germline removal and a great majority of pro-longevity interventions alter fat metabolism. Together, these observations suggest that autophagy is an effector of fat metabolism that might be critical for longevity^64^.

Although the results of our study apply to females, we do not expect significant differences between males/females in LCR or HCR. In fact, recent studies dedicated to assessing sex differences in these animals did not find them with respect to physical activity, weight, and response to calorie restriction^65^. The cross-sectional nature of our work introduces certain limitations associated with this approach regarding the inherent heterogeneity of animal populations, which is especially evident in middle-aged rats, in both HCR and LCR. These findings are not unique to rats but have been observed in other species, including humans, and may be given by individuals on the “right or wrong track of the aging process”. The heterogeneity among individuals decreases at young and old extremes of the aging process, as shown by respiration and metabolomics metrics. The decrease in heterogeneity at old age could be, in part, due to the prior die-off and depletion of the “poor agers” from the population being sampled.

Overall, the ensemble of data show that high running capacity rats after 35+ generations display enhanced cardiorespiratory performance sustained by higher mitochondrial health throughout the age range studied, compared to low-running capacity counterparts. Our integrated examination of the heart, liver, and serum metabolomes constitutes a body of work that goes substantially beyond what has been done before. This advances the aging field in showing that there is a fundamental resilience of the heart to stress that resides in mitochondrial function whose rate of decline with aging appears to segregate with intrinsic exercise performance, a story that had not been appreciated before. Based on our findings that mitochondrial health in cardiomyocytes is positively associated with longevity in rats with higher vs. lower intrinsic exercise capacity, we propose that maintenance of mitochondrial health and preservation of function positively affects the quality of aging and longevity across taxa.

## Methods

### Experimental model details

The present study utilized two lines of female rats termed HCRs and LCRs selected for running capacity in a treadmill under controlled acceleration. The highest and lowest runners, 1 for each sex from each of 13 families entered a rotational breeding scheme^38^. The running capacity phenotype was maintained over at least 28 generations accompanied by progressively increasing between-line differentiation and decreasing within-line diversity, indicating substantial genomic evolution^42^. In this work, the HCR and LCR rats were from generations 35 to 38 and were obtained from Drs. Lauren G. Koch and Steven L. Britton at The University of Toledo College of Medicine and Life Sciences, Toledo, OH, where all procedures were approved by the University of Toledo Institutional Animal Care and Use Committee in accordance with the National Institutes of Health Guide for the Care and Use of Laboratory Animals. Animals were housed two or three per cage with same-sex littermates in specific pathogen-free facilities in a controlled 12 : 12 h light–dark cycle with the light cycle occurring in the daytime and a target room temperature of 22 °C. All animals were fed a rodent pellet diet (Labdiet #5001; Purina Mills, Richmond, IN) and water was provided ad libitum.

The N/NIH HS utilized in the present study was obtained from Dr. Solberg Woods, L. at the Medical College of Wisconsin^66^. The N/NIH rats were one month of age when they came from Wisconsin and were grown until 6 months old at the National Institute on Aging. All procedures were approved by the National Institute on Aging Animal Care and Use Committee in accordance with the National Institutes of Health Guide for the Care and Use of Laboratory Animals. Animals were delivered to quarantine facility for prophylactic treatment for fur mites and maintained on Fenbendazole feed for two weeks prior to acceptance into vivarium. Once there, animals were housed two per cage with same-sex littermates in specific pathogen-free facilities in a controlled 12 : 12 h light–dark cycle with the light cycle occurring in the daytime and a target room temperature of 22 °C. All animals were fed a rodent pellet diet (Teklad, 2016SX Global 18% Protein Extruded Rodent Diet (Sterilizable)) and water was provided ad libitum until the time of killing at the age of 6 months.

### Method details

All the experiments described below, except for metabolomics, were performed following a blinding protocol, which was utilized throughout the study, and for all measurements. The identity of the rats (HS, HCR, LCR) was kept by the technician handling the animals/cells and was not revealed until after all the data were processed and analyzed by the investigators involved in the study. Therefore, any of the investigators collecting and analyzing data knew the identity of the animals until the end, including microscopy experiments (see below).

### Cardiomyocytes isolation and high-throughput respiratory measurements

Cardiomyocytes were isolated from 6-, 17-, or 24-month-old rats following standard procedures as described in ref. ^67^. Briefly, once isolated in Krebs Henseleit buffer (KHB) containing 1 mM CaCl_2_, the cardiomyocytes suspension was let to sediment twice, 5 min each at 1 × *g*, to separate viable from unviable (round “meat balls”) cells as the former sediment faster than the latter. The viable cardiomyocytes were recovered directly from the bottom of the tube and carefully pipette-transferred to Dulbecco’s modified Eagle’s medium (DMEM) supplemented with 1 g/l glucose, 100 units/ml penicillin, 100 μg/ml streptomycin, and 5% fetal bovine serum (Hyclone, Logan, UT) (DMEM medium), counted with a hemocytometer, and plated at a density of 500–4000 viable cells/well on a laminin covered multi-well plate. The laminin covered plates were incubated overnight, the day before the experiment, at room temperature with 0.01 mg/ml laminin solution freshly diluted in phosphate-buffered saline pH 7.4. The excess laminin solution was discarded shortly before seeding the cardiomyocytes. After seeding, the cells were maintained in DMEM medium at 37 °C in a humidified incubator with 5% CO_2_ and 95% O_2_ for 3 h to allow cells to attach to the laminin covered surface. Finally, the DMEM medium and dead cells (that do not attach) were removed by inverting the plate, the wells washed with KHB, followed by addition of 120 μl of KHB containing 1 mM glucose to each well for assaying OCR (final 80–85% cell viability, comparable between all experimental groups). We note that the approximate number of total cells recovered after the two sedimentation cycles were similar as age increased, and the recovery from the 3500–4000 cells per well initially plated, at the end of the attachment period, were not different as judged by the cell count performed after the OCR measurements. To avoid selection bias, isolations that rendered fewer viable cells than our standard 80–85% cell viability, were discarded.

Importantly, throughout the whole study, both LCR and HCR cardiomyocytes freshly isolated the same day were run paired in a 96-well plate for Rres, VO_2_max, and, always, processed by the same operators. This enabled us to lessen, e.g., technical, equipment, differences to interfere with the response of each strain’s cardiomyocytes. Importantly, the same protocol utilized for Rres, VO_2_max also applied for all other metrics as well, such as autophagy/mitophagy (confocal and EM), *t*_m_PTP, lipofuscin, which were obtained using a subset of freshly isolated cells from the same isolation batch (see “Confocal fluorescence imaging” and “Electron microscopy”).

The protocol for measuring respiration in isolated cardiomyocytes comprised high-throughput OCR quantification using a Seahorse XFe96 equipment, in the absence of substrate (baseline), followed by successive measurements performed on the same cells when exposed to subsequent additions of substrate (OCR_substr_), oligomycin (OCR_oligo_), FCCP (OCR_FCCP_), and respiratory inhibitors (antimycin + rotenone). Rres was quantified by subtracting OCR_substr_ from OCR_FCCP_ in the presence of either substrate or their combination (see Fig. 1a and Supplementary 1). VO_2_max was assigned to OCR_FCCP_ (Supplementary Fig. 2).

The abovementioned protocol carried out in the Seahorse XFe96 equipment, included three baseline measurements of the OCR, followed by OCR_substr_ comprising addition of 20 μl substrates’ stock solutions containing either glucose (Gluc, 4 mM final concentration + 1 mM already present in KHB = 5 mM) or Palm (200 μM bound to fatty acid-free bovine serum albumin (BSA), 4 : 1 Palm : BSA)^68^, or the combination Gluc + Palm at their respective concentrations^69^. Next, OCR_oligo_ was performed by adding 10 μM oligomycin followed by OCR_FCCP_ with 1.23 μM FCCP and 10 μM each of antimycin A/rotenone. Preliminary experiments were performed to demonstrate the linearity of the OCR response as a function of cell number and the expected behavior in the presence of the electron transport chain inhibitors, oligomycin, FCCP and antimycin A/rotenone^70^. At the end of the protocol, the cells were detached from the bottom of the plate by trypsin (0.05%) digestion for 10 min at 37 °C followed by addition of FBS and 4% formaldehyde to fix the cell for counting in a hemocytometer. The normalized OCR values are expressed in pmol or nmol O_2_/min/10^4^ cells.

### Mitochondrial isolation and high-throughput bioenergetic measurements

Procedures for the isolation and handling of mitochondria from rat hearts were performed as previously described^27^. Mitochondrial isolation was carried out in small (2–4 g) fresh heart tissue from 6-month-old rats, which were killed, the heart quickly excised, and the rest of the procedure was performed on ice. Briefly, the tissue was homogenized in the presence of 0.8 mg bacterial proteinase (type XXIV, Sigma), which was added just before starting the homogenization procedure, and the homogenate processed for mitochondrial isolation as previously described.

High-throughput Seahorse XFe96 OCR of isolated rat heart mitochondria under state 3 was determined in KCl-based assay medium in the presence of 5/5 mM G/M and 0.5 mM ADP. The oligomycin-sensitive OCR was obtained by subtracting from state 3 OCR the state 4 OCR measured in the presence of 10 μM oligomycin; *n* = 60/3 experiments, *P* < 0.0001.

The mitochondrial OCR was evaluated using the equivalent of 5 μg of mitochondrial protein/well plated on XF96 PET plates coated with polyethyleneimine, as previously described^69,71^. The assay was carried out under three different substrate conditions: 5 mM glucose, 20 μM PCoA, or both substrates together. Under β-oxidation fueled conditions in the presence of 20 μM PCoA, 0.5 mM malate and 0.5mM l-carnitine were also present, in a Respiration Assay Medium (200 μl final assay volume) containing (in mM): 137 KCl, 2 KH_2_PO4, 0.5 EGTA, 2.5 MgCl_2_, and 20 HEPES at pH 7.2 and 37 °C in the presence of 0.2% fatty acid-free BSA^71^. The OCR corresponding to states 4 and 3 respiration was determined before and after addition of 500 μM ADP, respectively. Respiratory Control Ratios (state 3/state 4) of 5 or higher were obtained with 5 mM/5 mM G/M. Then, 1 μM FCCP was added to uncouple respiration and, finally, 10 μM each of antimycin A and rotenone, to confirm the mitochondrial origin of the O_2_ consumption observed. The experimental rates are expressed in pmol min^−1^ mg^−1^ mitochondrial protein. As positive controls, samples were run in the absence of β-oxidation with NADH-linked substrates (G/M, 5/5 mM).

### Confocal fluorescence imaging

Isolated cardiomyocytes were resuspended in HEPES buffer: 137 mM NaCl, 4.9 mM KCl, 1.2 mM MgSO_4_, 1.2 mM NaH_2_PO_4_, 15 mM glucose, 20 mM HEPES, and 1.0 mM CaCl_2_ (pH 7.3). Lipofuscin autofluorescence was excited using 633 nm He-Ne laser; for all other confocal microscopy experiments, cardiomyocytes were loaded with an appropriate dye. Cells were imaged with an LSM-510 inverted confocal microscope, using a Zeiss Plan-Apochromat ×63/1.4 numerical aperture oil immersion objective (Carl Zeiss, Inc., Jena, Germany) with the optical slice set to 1 µm. Images were processed by MetaMorph software (Molecular Devices, San Jose, CA).

Imaging was performed with 15–20 randomly selected CytoID-loaded cardiomyocytes per cell batch isolation and analyzed using MetaMorph image analysis software. The % area occupied by the autophagolysosomes was referred to the whole cell area.

### Determination of mPTP-ROS threshold

Experiments were conducted as described previously^7^, using a method to quantify the ROS susceptibility for the induction of mPTP in individual mitochondria within cardiac myocytes^8^. Briefly, isolated cardiomyocytes were loaded with 100 nM tetramethylrhodamine methyl ester (TMRM; Invitrogen I34361) for at least 2 h at room temperature. Line scan images at 2 Hz were recorded from ~22 mitochondria arrayed along individual myofibrils with *λ*_exc_ at 543 nm and collecting *λ*_em_ at >560 nm. The confocal pinhole was set to obtain spatial resolutions of 0.4 µm in the horizontal plane and 1 µm in the axial dimension. Repetitive laser scanning of this row of mitochondria in a myocyte loaded with TMRM results in incremental, additive exposure of only the laser-exposed area to the photodynamic production of ROS and consequent mPTP induction, which can be clearly identified by the immediate dissipation of ΔΨ_m_ in individual mitochondria, visualized as a point in time where “columns” of the line scan image suddenly lose TMRM fluorescence becoming black (Fig. 3b). The ROS threshold for mPTP induction (*t*_mPTP_) was determined as the average time necessary to elicit the first ΔΨ_m_ depolarization in the exposed row of mitochondria (Fig. 3a, b).

### Autophagy

Cardiomyocytes were loaded with the autophagy dye from CYTO-ID Autophagy detection kit (ENZO 51031-K200), according the manufacturer protocol (dilution 1 : 500 in HEPES buffer) and incubated for 30 min at room temperature. The dye was washed out with HEPES buffer and cells imaged using *λ*_exc_ 488 nm and *λ*_em_ > 505 nm by confocal fluorescence microscopy (LSM-510 inverted confocal microscope).

### Electron microscopy

Cardiomyocytes pellets were processed for stereological analysis. Fixation for EM was performed using 2% glutaraldehyde–3% paraformaldehyde in 0.1 M sodium cacodylate buffer (0.1 M sucrose, 2 mM CaCl_2_). Samples were post fixed in 1% osmium tetroxide for 1 h at 4 °C in the same buffer, dehydrated, and then embedded in Embed 812 resin (Electron Microscopy Sciences, Hatfield, PA) through a series of resin resin-propylene oxide gradients to pure resin. Blocks were formed in fresh resin contained in silicon molds, and the resin was polymerized for 48–72 h at 65 °C. Ultrathin (40–60 nm) sections were stained with uranyl acetate and lead citrate, and then examined on a Jeol JEM1400 electron microscope (Servicio Centralizado de Apoyo a la Investigación, SCAI, University of Córdoba, Spain) by a blinded investigator (see above section: “Method details”). Micrographs at ×15,000 magnification were obtained from randomly selected areas of cardiomyocytes cytoplasm for quantitative analysis of autophagy/mitophagy figures. We determined two stereological parameters: (a) volume density of autophagy figures (Vv; i.e., the volume fraction of cardiomyocyte cytoplasm occupied by autophagy figures) and (b) numerical density of these figures (Nv, i.e., the number of autophagy figures per µm^3^ cell; see Fig. 2b). Volume density was obtained following a point analysis using a simple square lattice test system^72^. The numerical density was obtained using the formula

1 $$Nv = \frac{k}{\beta }\frac{{\mathrm {Na}^ {3/2}}}{{\mathrm {Vv}^{1/2}}}$$

where “Na” is the numerical profile density (number of autophagy figures/μm^2^ of cell), and *k* and *β* the autophagic figures size distribution and shape coefficient, respectively. These coefficients were calculated using the results of planimetric measurements in cardiomyocytes and assimilating figures shape to spheroids^72^. Planimetric measurements were performed using ImageJ software (NIH).

Autophagosomes were identified as double membrane vesicles with identifiable cargo and comparable density to the surrounded cytosol. Autolysosomes were denoted as single membrane vesicles containing non-identifiable cargo of density lower than the surrounded cytosol and fragmented organelles. Areas of vesicles and cytosol were measured using manual tracking with ImageJ software.

For mitochondrial metrics, only micrographs depicting longitudinal sections of cardiomyocytes with visible sarcomeres were utilized, and from each of these five to seven pictures were taken from four to six cells/fibers. From each picture, mitochondria were counted and measured to determine the number and area, doing the average of these metrics in each picture, and finally the average for each cell. With this procedure, a total of ~1600 up to ~2000 mitochondria per experimental group were counted/measured.

### Cross-sectional metabolomics in serum, heart, and liver tissue from LCR and HCR rats

Metabolomic analysis was performed by the West Coast Metabolomics Center at UC Davis (Davis, CA) in the heart, liver, and serum from nonfasted animals as previously described^73,74^. Data were acquired using the method as described by^75^, briefly summarized in ref. ^74^. Relative metabolite levels represent the MS peak amplitude normalized with respect to the total metabolites returned

### Bioinformatic and statistical analyses

MetaboAnalyst versions 3.0^76^ and 4.0^77^, an integrated web-based platform for comprehensive analysis of metabolomics data^78^ was utilized. Metabolites were normalized using the autoscaling function of MetaboAnalyst preceded by detection and removal of outliers. Univariate (analysis of variance), clustering (heat map, correlation matrix, and pattern search) and multivariate (PLSD) among other statistical analyses were applied to the metabolite profiles. According to PLSD and TWA analyses, we determined the metabolites responsible for the separation between groups as indicated in the main text. In metabolomics data, outliers were excluded from the statistics when above or below 1.5 times the interquartile range comprised between the 75% and 25% percentiles, respectively^79^. Shared and unique metabolites among strains, age, and organ were determined according to Venn diagrams. The groups of significantly shared metabolites under the corresponding conditions were analyzed with the “Pathways Analysis” module of MetaboAnalyst. Metabolic pathways were considered significantly enriched at log *p* ≥ 1.3 (*p* ≤ 0.05) and accordingly ranked.

TWA (Prism 8.0, GraphPad Software, San Diego, CA, USA) was performed with Sidak’s or Tukey’s multi-comparison test for age vs. strain or vs. substrate and their respective interaction, at each of the three ages (6-, 17-, and 24 months) (Figs 1–3 and Supplementary Figs 1–9). For individual metabolites, TWA (Prism 8.0, GraphPad Software, San Diego, CA, USA) was performed with Tukey’s multi-comparison test for strain, age, and strain/age interaction, in serum and each organ (heart and liver). The statistical significance of the effect of strain, age, and strain/age interaction were evaluated for metabolites that exhibited at least one significant change according to Tukey’s multi-comparison test for either factor (Fig. 6a). Venn diagrams were plotted with Microcal Origin 9.5.

## Supplementary information

Supplemental_Final

Report summary

## Data Availability

The metabolomics data of the present work is available at the NIH Common Fund’s National Metabolomics Data Repository (NMDR) website, the Metabolomics Workbench (https://www.metabolomicsworkbench.org) where it has been assigned Project ID (PR001027). The data can be accessed directly via Project DOI: (10.21228/M8Z41B). This work is supported by NIH grant U2C-DK119886. Further information and requests for resources and reagents should be directed to and will be fulfilled by Steven J. Sollott (sollotts@grc.nia.nih.gov) and Miguel A. Aon (miguel.aon@nih.gov).
