# Supplementary material for: Mitochondrial health is enhanced in rats with higher vs. lower intrinsic exercise capacity and extended lifespan
Source: NPJ Aging Mech Dis. 2021 Jan 4;7:1. doi: 10.1038/s41514-020-00054-3 (PMC7782588; doi:10.1038/s41514-020-00054-3)
Supplement: Supplementary file 1 — Supplemental_Final [file 41514_2020_54_MOESM1_ESM.pdf]

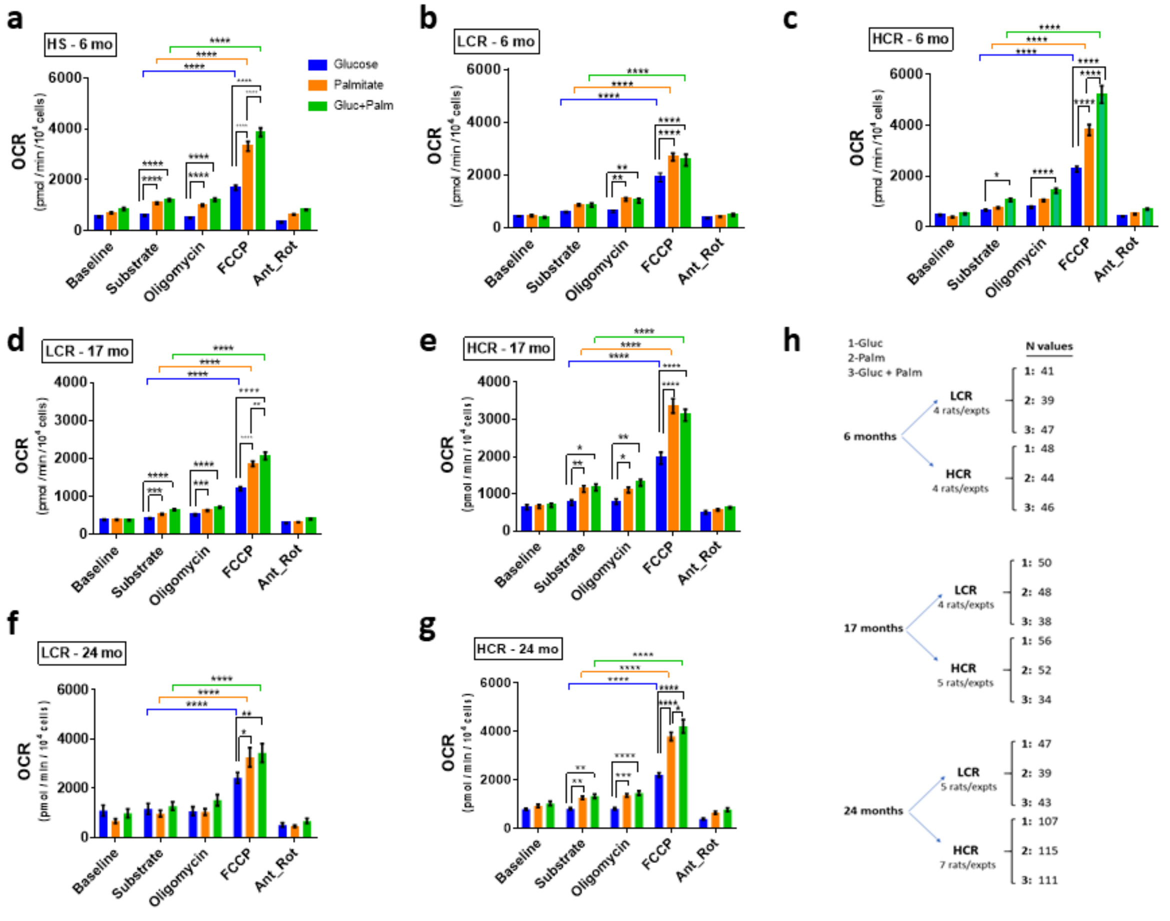


Supplementary Figure 1 related to Fig. 1a

**Supplementary Fig. 1 related to Fig. 1a. High throughput Oxygen Consumption Rate (OCR) in cardiomyocytes from 6-, 17-, and 24-months old LCR/HCR rats, and 6-months old HS**

The OCR was measured in a Seahorse equipment at each step of the protocol utilized, according to our experimental design (Fig. 1a, inset) as described in Methods, section: *Cardiomyocytes isolation and high throughput respiratory measurements.* Panel H shows full experimental design and experiments/*n* values for LCR/HCR, and in legend of Figure 1 for HS.

In all experiments, LCR (panels b, d, f) and HCR (panels c, e, g) cardiomyocytes were isolated the same day of the experiment and assayed paired in a 96-well plate. The respiratory reserve (Rres) - defined as the difference between the uncoupled $OCR_{FCCP}$ and the $OCR_{substr}$, was determined for each strain and two different substrates, glucose (Gluc, 5mM) or palmitate (Palm, 0.2mM) bound to fatty-acid free bovine serum albumin, 4:1, or the combination of both substrates, at their respective concentrations (see legend Fig. 1, and Methods, section: *Cardiomyocytes isolation and high throughput respiratory measurements*). The normalized OCR values, expressed in pmol $O_2$ / min / $10^4$ cells, correspond to averages of 9-22 technical replicates in each experiment. See also Supplementary Figure 4 for representative examples of raw OCR data (pmol/min) across the age span studied.

In all cases, data are represented as mean ± SEM. The statistical significance is indicated by *, p<0.05; **, p<0.01; ***, p<0.001; ****, p<0.0001; NS, not significant.

## MAXIMAL RESPIRATORY CAPACITY ($VO_2$ max)

**a**

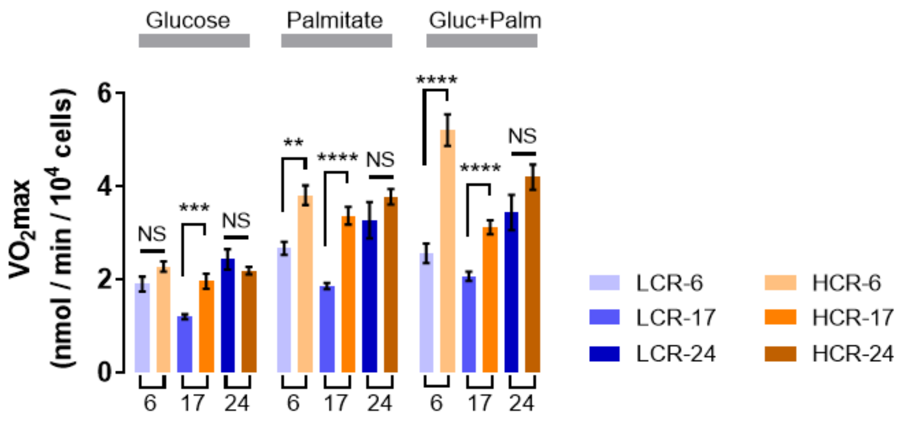


**b**

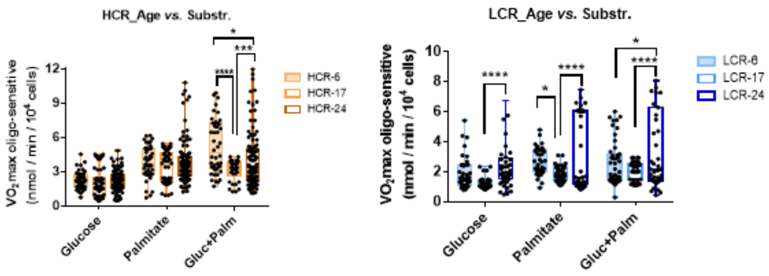


| | HCR - $VO_2$ max_Age vs Substr. | | |
|---|---|---|---|
| Source of Variation | % of total variation | P value | P value summary |
| Interaction | 2.06 | 0.0026 | ** |
| Substrate | 16.9 | < 0.0001 | **** |
| Age | 2.81 | < 0.0001 | **** |

| | LCR - $VO_2$ max_Age vs Substr. | | |
|---|---|---|---|
| Source of Variation | % of total variation | P value | P value summary |
| Interaction | 0.214 | 0.9084 | ns |
| Substrate | 5.91 | < 0.0001 | **** |
| Age | 11.9 | < 0.0001 | **** |

Supplementary Figure 2 related to Fig. 1a

**Supplementary Fig. 2 related to Fig. 1a. Maximal respiratory capacity ($VO_2$max) in isolated cardiomyocytes from HCR and LCR as a function of substrate and age**

(a) The $VO_2$max of cardiac myocytes was measured as the maximal OCR in the presence of 1.23μM FCCP ($OCR_{FCCP}$) (see legend Fig. 1, and Methods, section: *Cardiomyocytes isolation and high throughput respiratory measurements*). Panel h in Supplementary Figure 1 informs experiments/*n* values for $VO_2$max from LCR/HCR.

(b) Two-way ANOVA (TWA) of $VO_2$max data from HCR (left panel) and LCR (middle panel) using age and substrate as factors and Tukey's multiple comparison test (GraphPad Prism 8.0). The tabular results of TWA including *p* values are depicted on the right panel. Displayed are all data points.

In all cases, data are represented as mean ± SEM. The statistical significance is indicated by *, $p<0.05$; **, $p<0.01$; ***, $p<0.001$; ****, $p<0.0001$; NS, not significant.

# RESPIRATORY RESERVE (Rres)

## AGE *vs.* SUBSTRATE

**a**

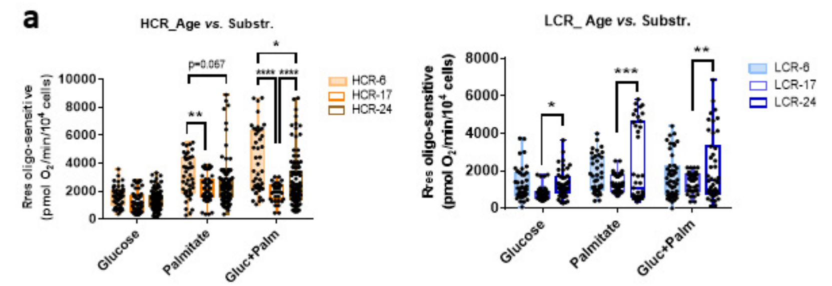


## TWO WAY ANOVA

| | HCR - Rres_Age vs Substr | | |
|---|---|---|---|
| Source of Variation | % of total variation | P value | P value summary |
| Interaction | 2.86 | 0.0001 | *** |
| Substrate | 15.1 | < 0.0001 | **** |
| Age | 6.43 | < 0.0001 | **** |

| | LCR - Rres_Age vs Substr | | |
|---|---|---|---|
| Source of Variation | % of total variation | P value | P value summary |
| Interaction | 0.706 | 0.5326 | ns |
| Substrate | 6.85 | < 0.0001 | **** |
| Age | 6.78 | < 0.0001 | **** |

**b**

## AGE *vs.* STRAIN

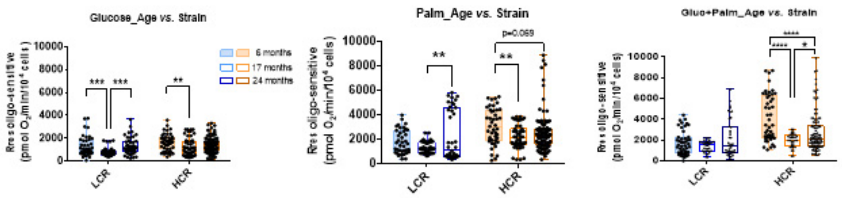


| | Glucose - Rres_ Age vs Strain | | |
|---|---|---|---|
| Source of Variation | % of total variation | P value | P value summary |
| Interaction | 0.915 | 0.1731 | ns |
| Strain | 3.16 | 0.0005 | *** |
| Age | 7.04 | < 0.0001 | **** |

| | Palmitate - Rres_ Age vs Strain | | |
|---|---|---|---|
| Source of Variation | % of total variation | P value | P value summary |
| Interaction | 1.92 | 0.0275 | * |
| Strain | 6.78 | < 0.0001 | **** |
| Age | 4 | 0.0006 | *** |

| | Gluc+Palm - Rres_ Age vs Strain | | |
|---|---|---|---|
| Source of Variation | % of total variation | P value | P value summary |
| Interaction | 5.18 | < 0.0001 | **** |
| Strain | 9.04 | < 0.0001 | **** |
| Age | 5.98 | < 0.0001 | **** |

Supplementary Figure 3 related to Fig. 1a

**Supplementary Fig. 3 related to Fig. 1a. Two-way ANOVA (TWA) of Rres in isolated cardiomyocytes from HCR and LCR as a function of substrate, age, and strain**

(a) TWA of Rres data from HCR (left panel) and LCR (middle panel) using age and substrate as factors and Tukey's multiple comparison test (GraphPad Prism 8.0). The tabular results of TWA including *p* values are depicted on the right panel. Displayed are all data points.

(b) TWA of Rres data from HCR and LCR using age and strain as factors in glucose (left panel), palmitate (Palm, middle panel) and their combination (Gluc + Palm, right panel). The tabular results of TWA using Tukey's multiple comparison test (GraphPad Prism 8.0), including *p* values, are depicted on the right. Displayed are all data points.

Experiments / n values for Rres from LCR/HCR are described in legends of Figures 1 and Supplementary 1.

In all cases, data are represented as mean ± SEM. The statistical significance is indicated by *, p<0.05; **, p<0.01; ***, p<0.001; ****, p<0.0001; NS, not significant.

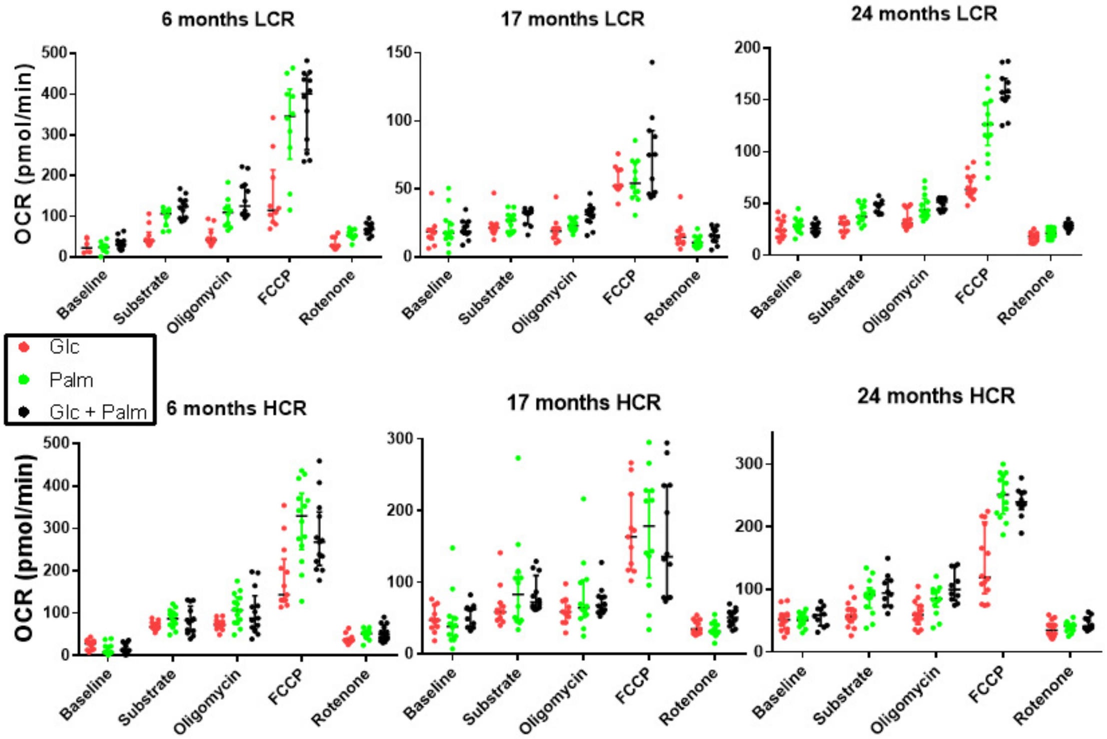


Supplementary Figure 4 related to Fig. 1a

**Supplementary Fig. 4 related to Fig. 1a. High throughput raw OCR in cardiomyocytes from 6-, 17-, and 24-months old LCR/HCR rats**

Representative examples of raw (non-normalized) OCR measured in a Seahorse equipment of cardiomyocytes from LCR (top panels) and HCR (bottom panels) at 6, 17, and 24 months of age, 3 substrates, and each step of the protocol utilized (Fig. 1a, inset), under the conditions described in the legends of Figs. 1 and Supplementary 1 (see also Methods, section: *Cardiomyocytes isolation and high-throughput respiratory measurements*). Depicted are the data points corresponding to technical replicates from paired OCR measurements of LCR and HCR cardiomyocytes in a 96-well plate at each of the indicated steps of the protocol.

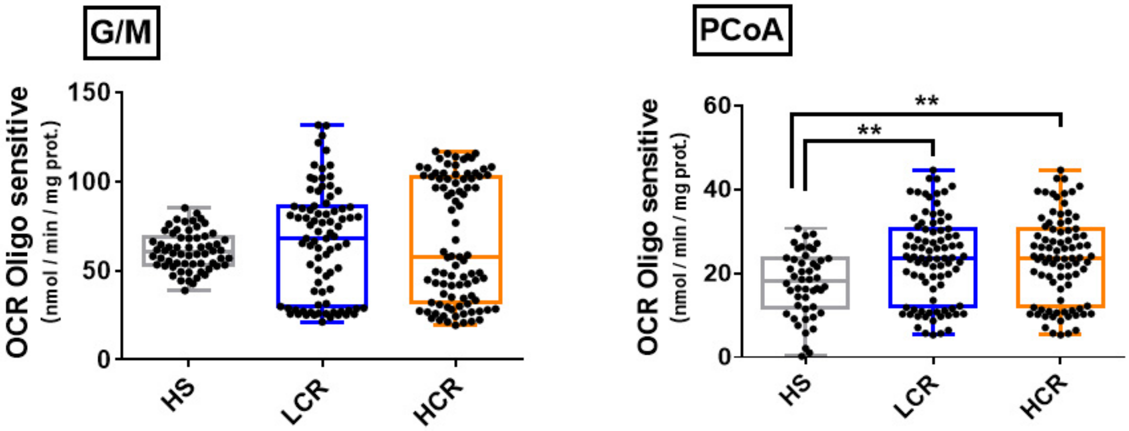


Supplementary Figure 5 related to Fig. 1b

**Supplementary Fig. 5 related to Fig. 1b. High throughput measurements of OCR in isolated mitochondria from LCR, HCR and HS hearts at 6 months of age**

OCR-oligomycin sensitive measured in a Seahorse equipment with mitochondria isolated from hearts of 6 months old HS, LCR or HCR rats, assayed in the presence of glutamate/malate (G/M) or palmitoyl CoA (PCoA)/malate as detailed in Methods, section: *Mitochondrial isolation and high throughput bioenergetic measurements*. Displayed are the normalized OCR data points; experiments/*n* values are described in the legend of Figure 1b. The statistical analysis corresponds to one-way ANOVA with Tukey's multiple comparison test (GraphPad Prism 8.0). In all cases, data are represented as mean ± SEM. The statistical significance is indicated by *, $p<0.05$; **, $p<0.01$; ***, $p<0.001$; ****, $p<0.0001$; NS, not significant.

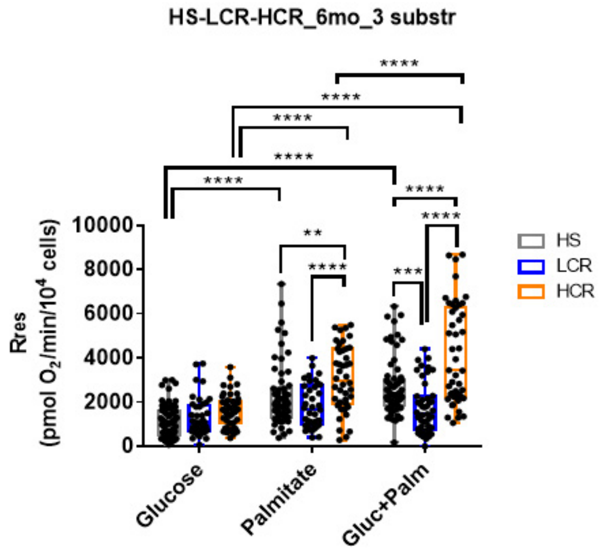


| | HS-LCR-HCR vs Substrate | | |
|---|---|---|---|
| | | | |
| Source of Variation | % of total variation | P value | P value summary |
| Interaction | 4.67 | < 0.0001 | **** |
| Strain | **16** | < 0.0001 | **** |
| Substrate | **11.4** | < 0.0001 | **** |

Supplementary Figure 6 related to Fig. 1c

**Supplementary Fig. 6 related to Fig. 1c. Two-way ANOVA (TWA) of Rres in isolated cardiomyocytes from HCR, LCR and HS as a function of substrate and strain at 6 months of age**

(A) TWA of Rres data from HS, LCR and HCR at 6 months of age, using strain and substrate as factors, and Tukey's multiple comparison test (GraphPad Prism 8.0). The tabular results of TWA including *p* values are depicted on the right panel. Displayed are all data points of normalized Rres.

Experiments / *n* values for Rres from HS, LCR, and HCR are described in legends of Figures 1 and Supplementary 1.

In all cases, data are represented as mean ± SEM. The statistical significance is indicated by *, $p<0.05$; **, $p<0.01$; ***, $p<0.001$; ****, $p<0.0001$; NS, not significant.

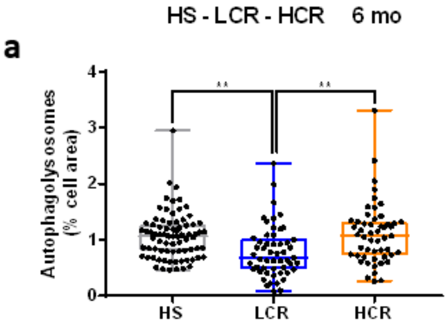


| | HS LCR HCR | |
|---|---|---|
| | | |
| Tukey's multiple comparisons test | Adjusted P Value | Summary |
| LCR vs. HCR | 0.0019 | ** |
| LCR vs. HS | 0.0024 | ** |
| HCR vs. HS | 0.9214 | ns |

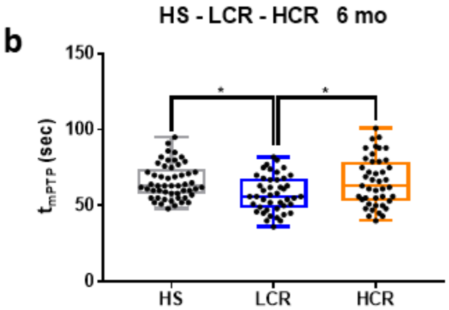


| | HS LCR HCR | |
|---|---|---|
| | | |
| Tukey's multiple comparisons test | Adjusted P Value | Summary |
| LCR vs. HCR | 0.0125 | * |
| LCR vs. HS | 0.0101 | * |
| HCR vs. HS | 0.9979 | ns |

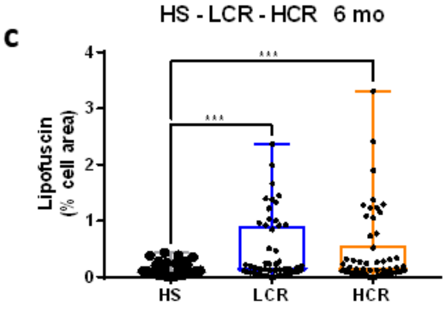


| | HS LCR HCR | |
|---|---|---|
| | | |
| Tukey's multiple comparisons test | Adjusted P Value | Summary |
| LCR vs. HCR | 0.9979 | ns |
| LCR vs. HS | 0.0002 | *** |
| HCR vs. HS | 0.0002 | *** |

Supplementary Figure 7 related to Fig. 1d

**Supplementary Fig. 7 related to Fig. 1d. One-way ANOVA of confocal live cell imaging of autophagy, mitochondrial fitness, and lipofuscin in isolated cardiomyocytes of HS, LCR and HCR at 6 months of age**

Cardiomyocytes isolated from 6 months old HS, LCR or HCR rats were subjected to determinations of (a) autophagy (HS: 3 rats/experiments, n=66-72; LCR: 4 rats/experiments, n=37-47; HCR: 4 rats/experiments, n=37-48); (b) $t_m$PTP (HS: 3 rats/experiments, n=52; LCR: 4 rats/experiments, n=52; HCR: 4 rats/experiments, n=53), and (c) lipofuscin (HS: 3 rats/experiments, n=78; LCR: 4 rats/experiments, n=52; HCR: 4 rats/experiments, n=53), as described in Methods, section: "*Confocal imaging and electron microscopy*..". The statistical analysis shown in tabular form on the right of panels a-c, corresponds to one-way ANOVA with Tukey's multiple comparison test (GraphPad Prism 8.0). Shown are all data points.

In all cases, data are represented as mean ± SEM. The statistical significance is indicated by *, $p<0.05$; **, $p<0.01$; ***, $p<0.001$; ****, $p<0.0001$; NS, not significant.

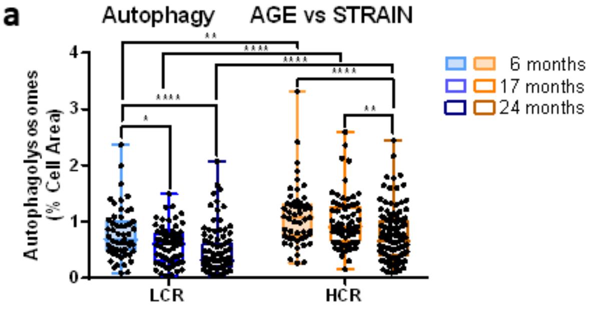


| | Autophagy - Age vs Strain | | |
|---|---|---|---|
| Source of Variation | % of total variation | P value | P value summary |
| Interaction | 0.24 | 0.5239 | ns |
| Strain | 11.1 | <0.0001 | **** |
| Age | 7.85 | <0.0001 | **** |

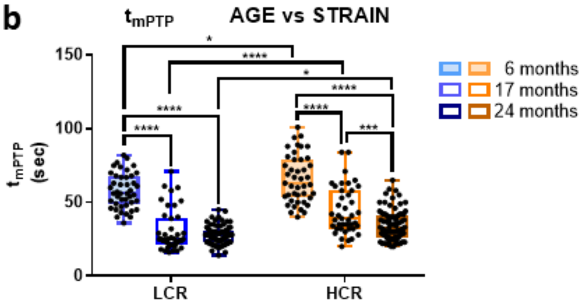


| | $t_{mPTP}$ - Age vs Strain | | |
|---|---|---|---|
| Source of Variation | % of total variation | P value | P value summary |
| Interaction | 0.666 | 0.0846 | ns |
| Strain | 6.15 | <0.0001 | **** |
| Age | 50.6 | <0.0001 | **** |

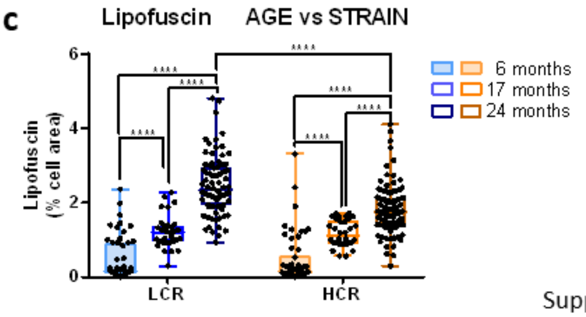


| | Lipofuscin - Age vs Strain | | |
|---|---|---|---|
| Source of Variation | % of total variation | P value | P value summary |
| Interaction | 2.42 | <0.0001 | **** |
| Strain | 1.14 | 0.0025 | ** |
| Age | 55.5 | <0.0001 | **** |

Supplementary Figure 8 related to Fig. 2a and Fig. 3a, c

**Supplementary Fig. 8 related to Fig. 2a and Fig. 3 a, c. TWA of autophagy, mitochondrial fitness and lipofuscin in isolated cardiomyocytes from LCR and HCR at 6, 17 and 24 months of age**
Cardiomyocytes isolated from LCR or HCR rats were subjected to determinations of (a) autophagy (see legend of Fig. 2a for experiments/*n* values); (b) $t_{m}$PTP (see legend of Fig. 3a for experiments/*n* values); and (c) lipofuscin (see legend of Fig. 3c for experiments/*n* values), as described in Methods, section: "*Confocal imaging and electron microscopy*…". The statistical analysis shown in tabular form on the right of panels a-c, corresponds to TWA with Tukey's multiple comparison test (GraphPad Prism 8.0). Shown are all data points.
In all cases, data are represented as mean ± SEM. The statistical significance is indicated by *, p<0.05; **, p<0.01; ***, p<0.001; ****, p<0.0001; NS, not significant.

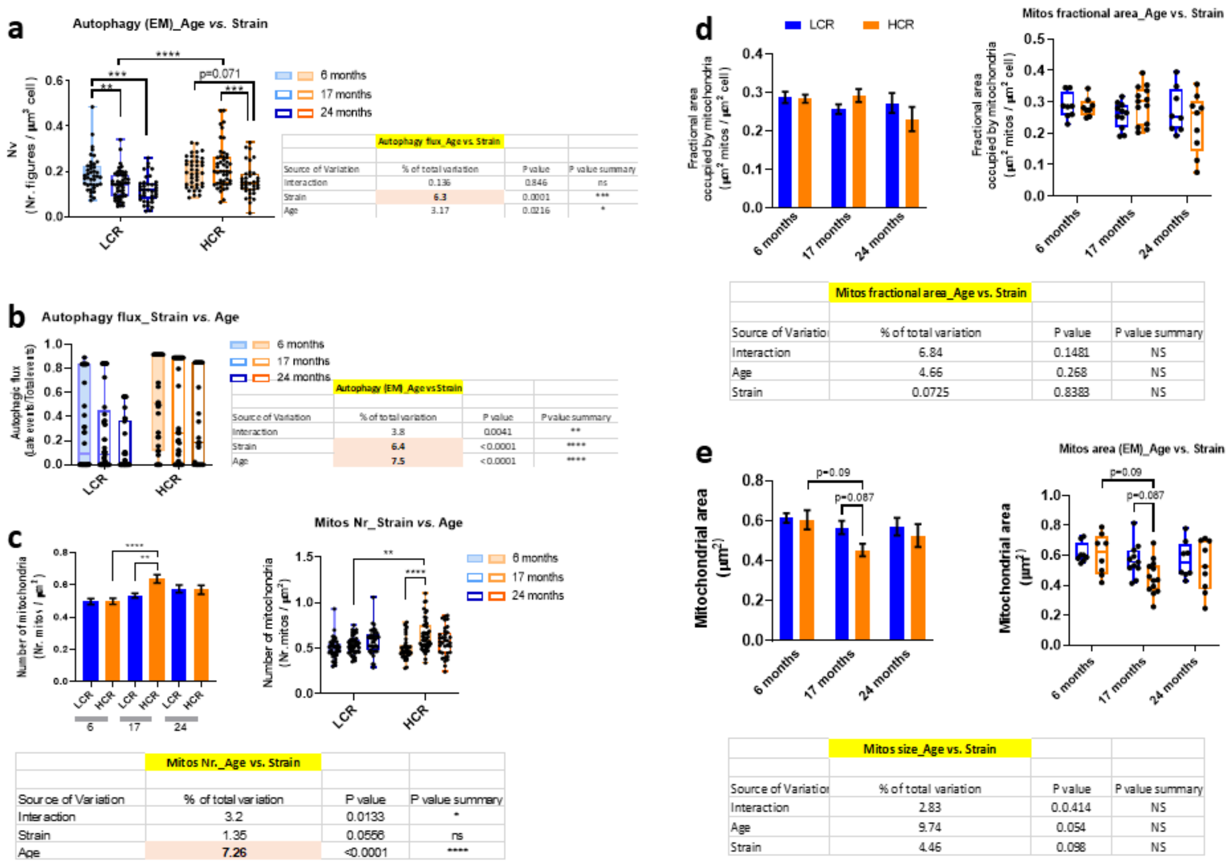


Supplementary Figure 9 related to Fig. 2b, c, e, f

**Supplementary Fig. 9 related to Fig. 2b, c, e, f. Two-way ANOVA (TWA) of EM autophagy/mitophagy, autophagic flux and mitochondrial mass metrics in isolated cardiomyocytes from LCR and HCR at 6, 17 and 24 months of age**

(a) Quantitation of total autophagy/mitophagy figures (autophagosomes [early] + autophagolysosomes [late]) observed by EM and normalized with respect to the cell volume as described in the legend of Figure 2b, and in Methods (section: *Confocal imaging and electron microscopy*…). All data points are shown.

(b) Quantitation of autophagic flux ([late] / [early + late]) figures (all data points) observed by EM in samples of LCR and HCR cardiomyocytes as described in the legend of Fig. 2c together with experiments/*n* values.

(c) Quantitation of the number of mitochondria in cardiomyocytes from 6- (LCR: 2 rats/experiments, n=40; HCR: 2 rats/experiments, n=40), 17- (LCR: 2 rats/experiments, n=48; HCR: 2 rats/experiments, n=47), and 24- (LCR: 2 rats/experiments, n=37; HCR: rats/experiments, n=35) months old rats.

In panels a-c, TWA with Tukey's multiple comparison test (GraphPad Prism 8.0) was performed; all data points are shown.

(d, e) Fractional area occupied by mitochondria (d) and mitochondrial area (e) were quantified from EM micrographs. For these determinations, only micrographs depicting longitudinal sections of cardiomyocytes with visible sarcomeres were utilized, and from each of these 5-7 pictures were taken from 4-6 cells/fibers. From each picture, mitochondria were counted and measured to determine number and area, doing the average of these metrics in each picture, and finally the average for each cell. With this procedure, a total of ~1600 up to ~2000 mitochondria per experimental group were counted/measured. In panels d, e, TWA with Sidak's multiple comparison test (GraphPad Prism 8.0) was performed; all data points are shown. In all cases, data are represented as mean ± SEM. The statistical significance is indicated by *, p<0.05; **, p<0.01; ***, p<0.001; ****, p<0.0001; NS, not significant.

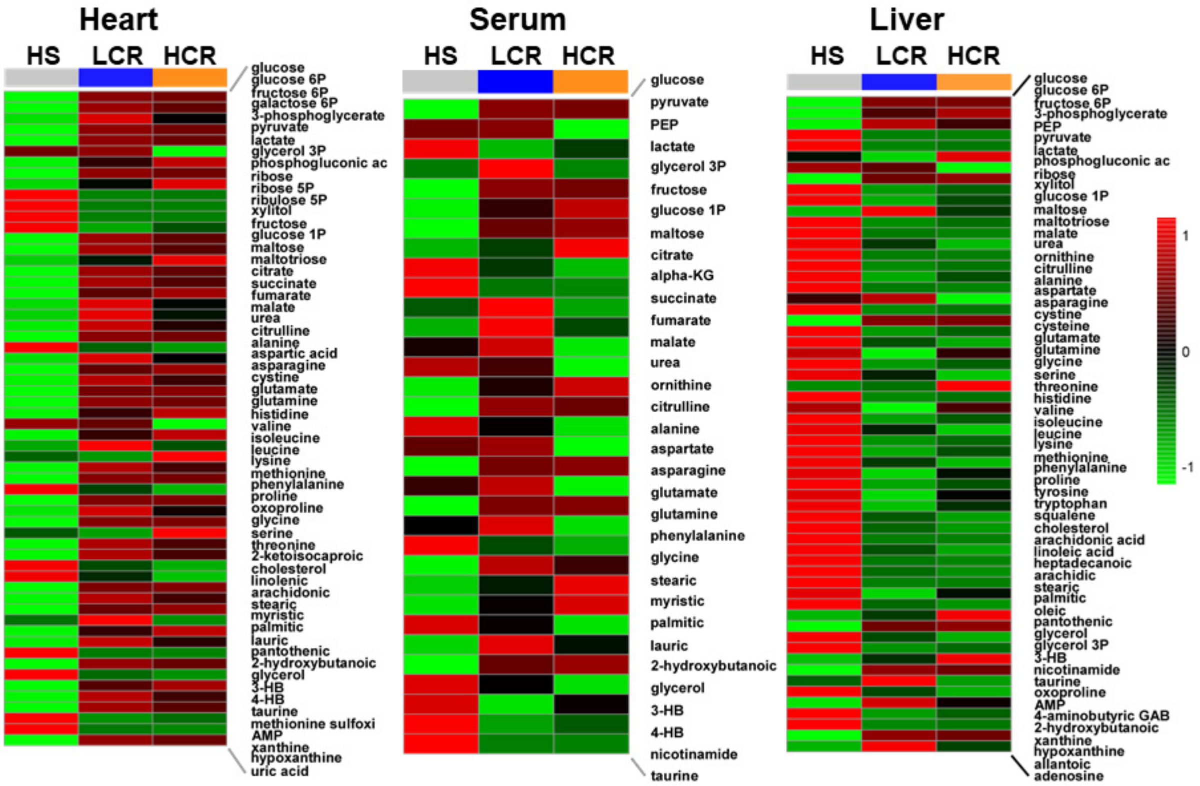


Supplementary Figure 10 related to Fig. 4b

**Supplementary Fig. 10 related to Fig. 4b. Average heat maps of significantly changed metabolites of heart, serum, and liver from HS, LCR and HCR**

Heat maps depict the relative metabolite levels in pseudo color denoting accumulation (in red) or depletion (in green) according to the scale on the right of the maps, in serum (middle), and heart (left) and liver (right) tissue from HS (left lane), LCR (middle lane) and HCR (right lane).

The metabolites are shown on the right of the heat maps, whereas the metabolic pathways to which the metabolites belong are shown in Figure 4b.

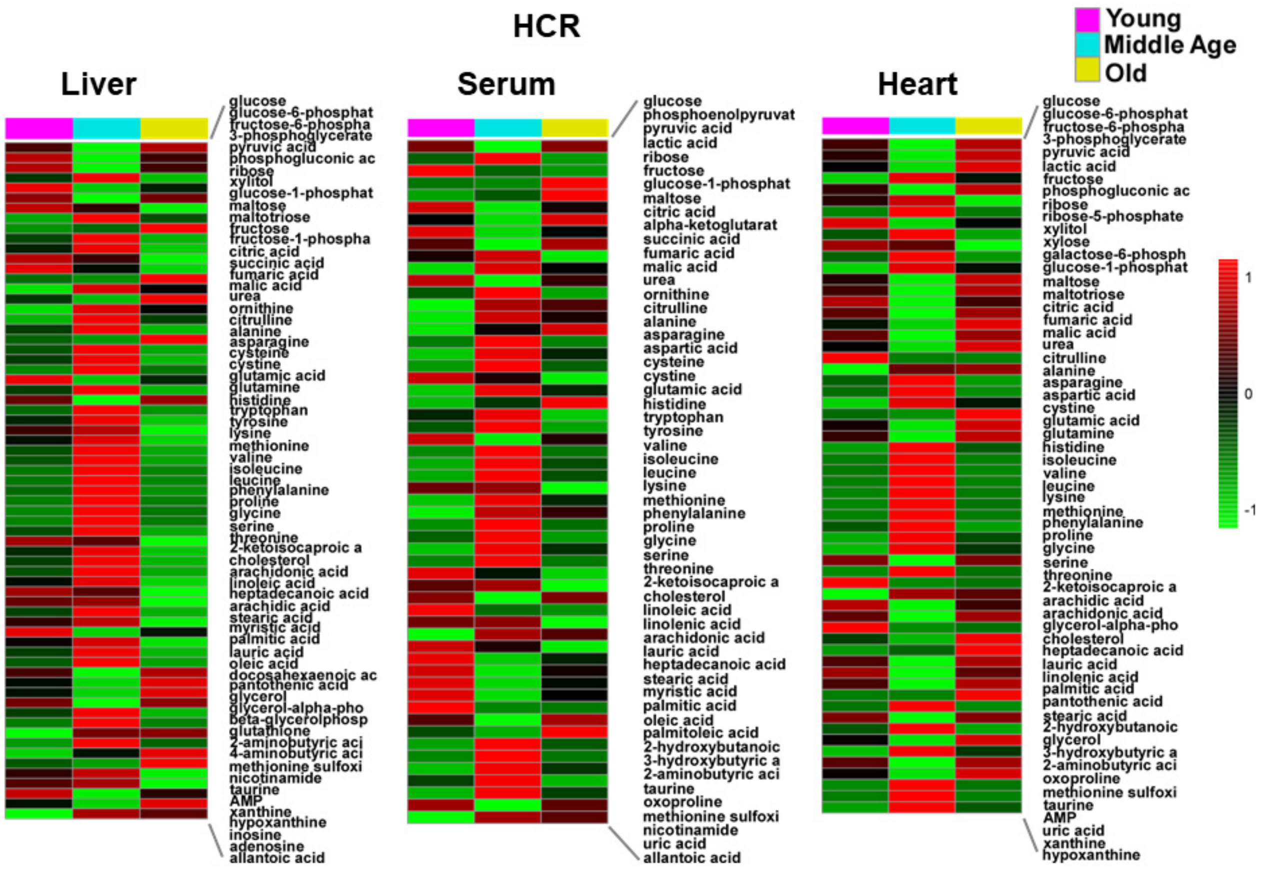


Supplementary Figure 11 related to Fig. 5a

**Supplementary Fig. 11 related to Fig. 5a. Average heat maps of significantly changed metabolites in heart, liver, and serum from HCR as a function of age**

Heat maps depict the relative metabolite levels in pseudo color denoting accumulation (in red) or depletion (in green) according to the scale on the right of the maps, in serum (middle), and liver (left) and heart (right) tissue from young (left lane), middle age (middle lane) and old (right lane) HCR. The metabolites are shown on the right of the heat maps, whereas the metabolic pathways to which the metabolites belong are shown in Figure 5a.

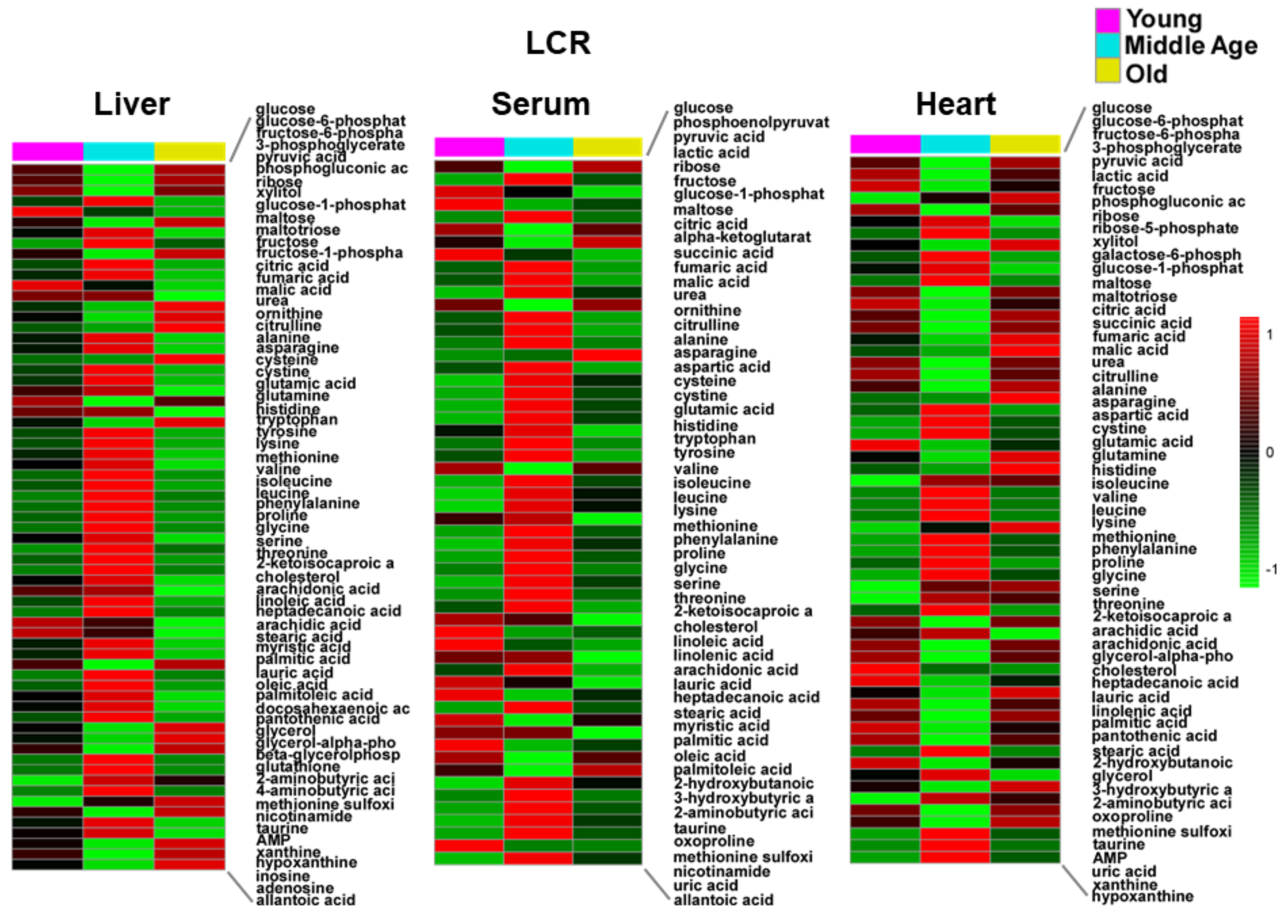


Supplementary Figure 12 related to Fig. 5b

**Supplementary Fig. 12 related to Fig. 5b. Average heat maps of significantly changed metabolites in heart, liver, and serum from LCR as a function of age**

Heat maps depict the relative metabolite levels in pseudocolor denoting accumulation (in red) or depletion (in green) according to the scale on the right of the maps, in serum (middle), and liver (left) and heart (right) tissue from young (left lane), middle age (middle lane) and old (right lane) LCR. The metabolites are shown on the right of the heat maps, whereas the metabolic pathways to which the metabolites belong are shown in Figure 5b.

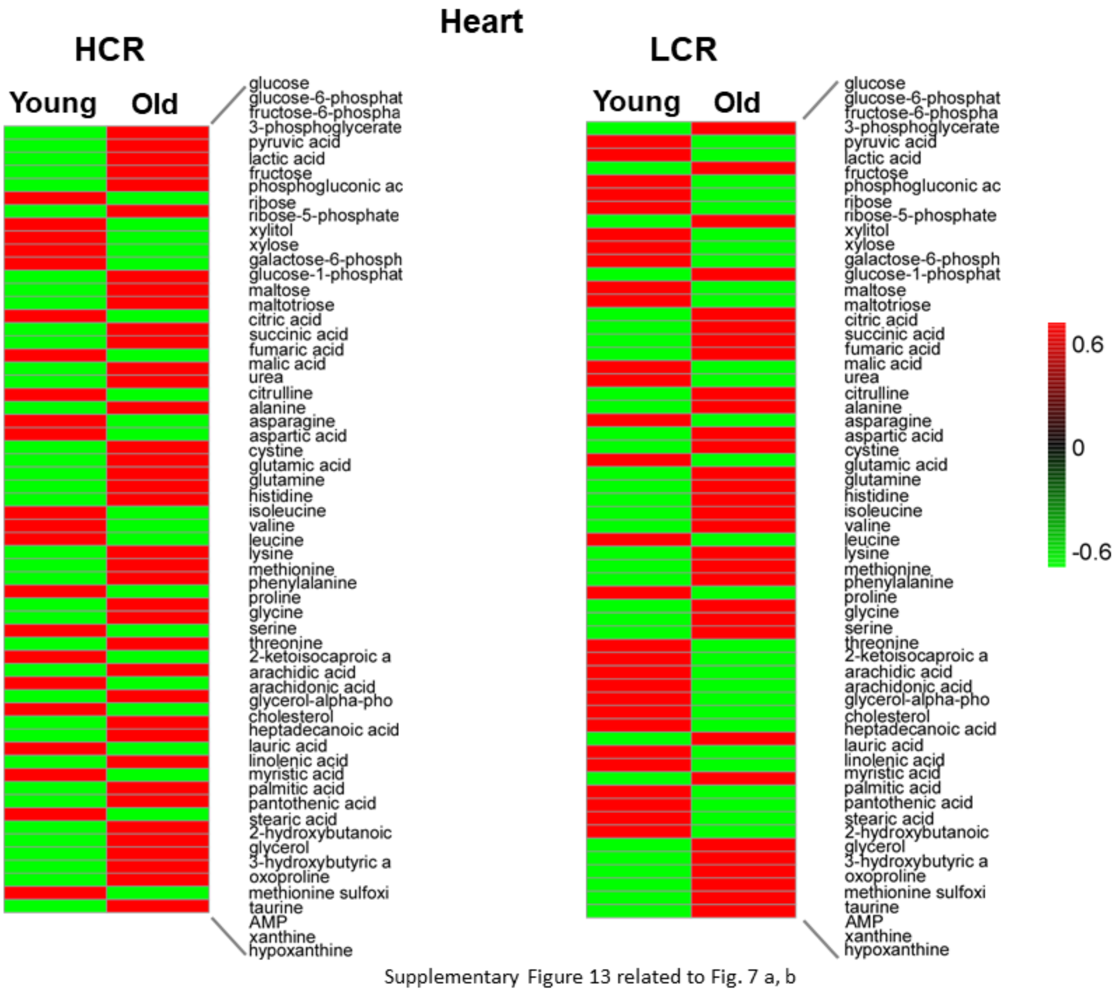


**Supplementary Fig. 13 related to Fig. 7 a, b. Comparative metabolomics of HCR *vs.* LCR, young *vs.* old, in heart**

Displayed are the average heatmaps of significantly changed metabolites in heart from HCR (left) and LCR (right) that identify the metabolites depicted in the heat maps of Fig. 7 a, b.

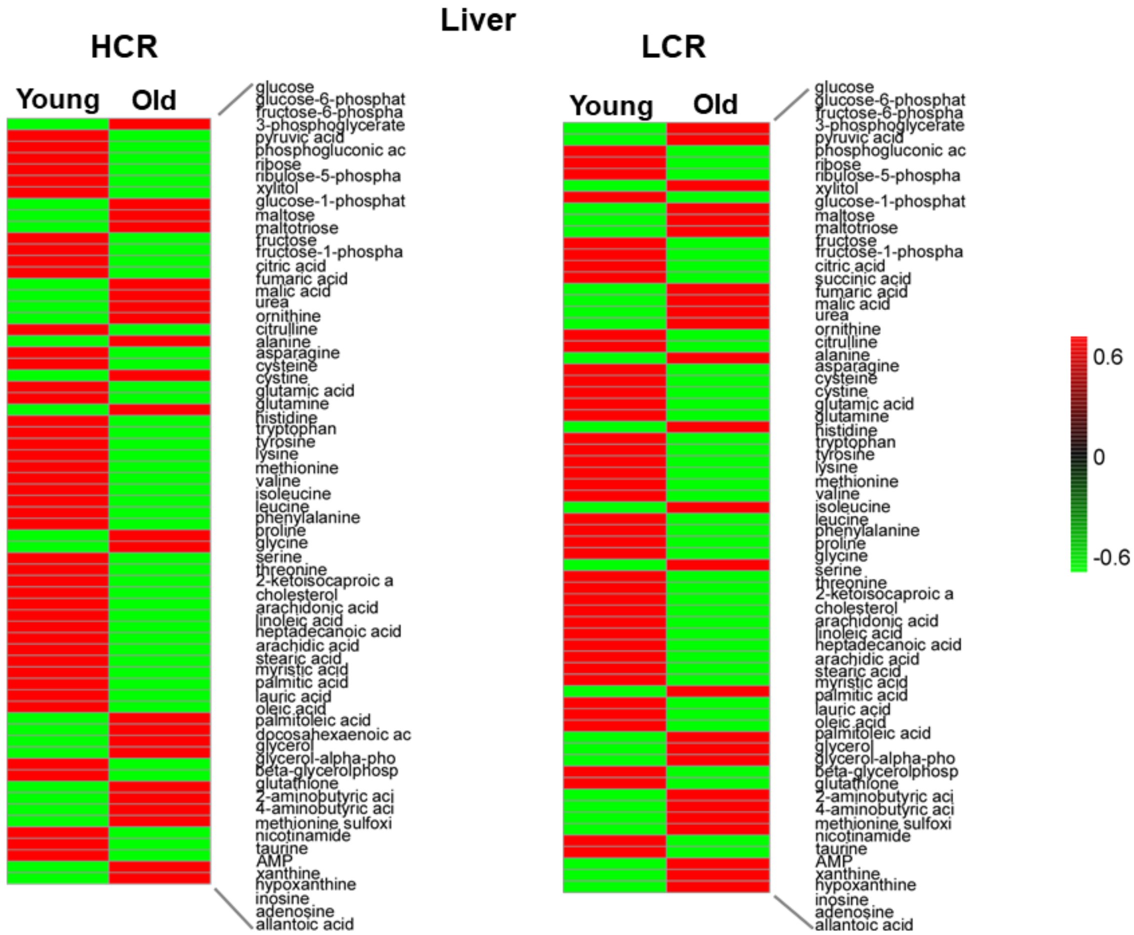


Supplementary Figure 14 related to Fig. 7 c, d

**Supplementary Fig. 14 related to Fig. 7 c, d. Comparative metabolomics of HCR *vs.* LCR, young *vs.* old, in liver**

Displayed are the average heatmaps of significantly changed metabolites in liver from HCR (left) and LCR (right) that identify the metabolites depicted in the heat maps of Figure 7 c, d.

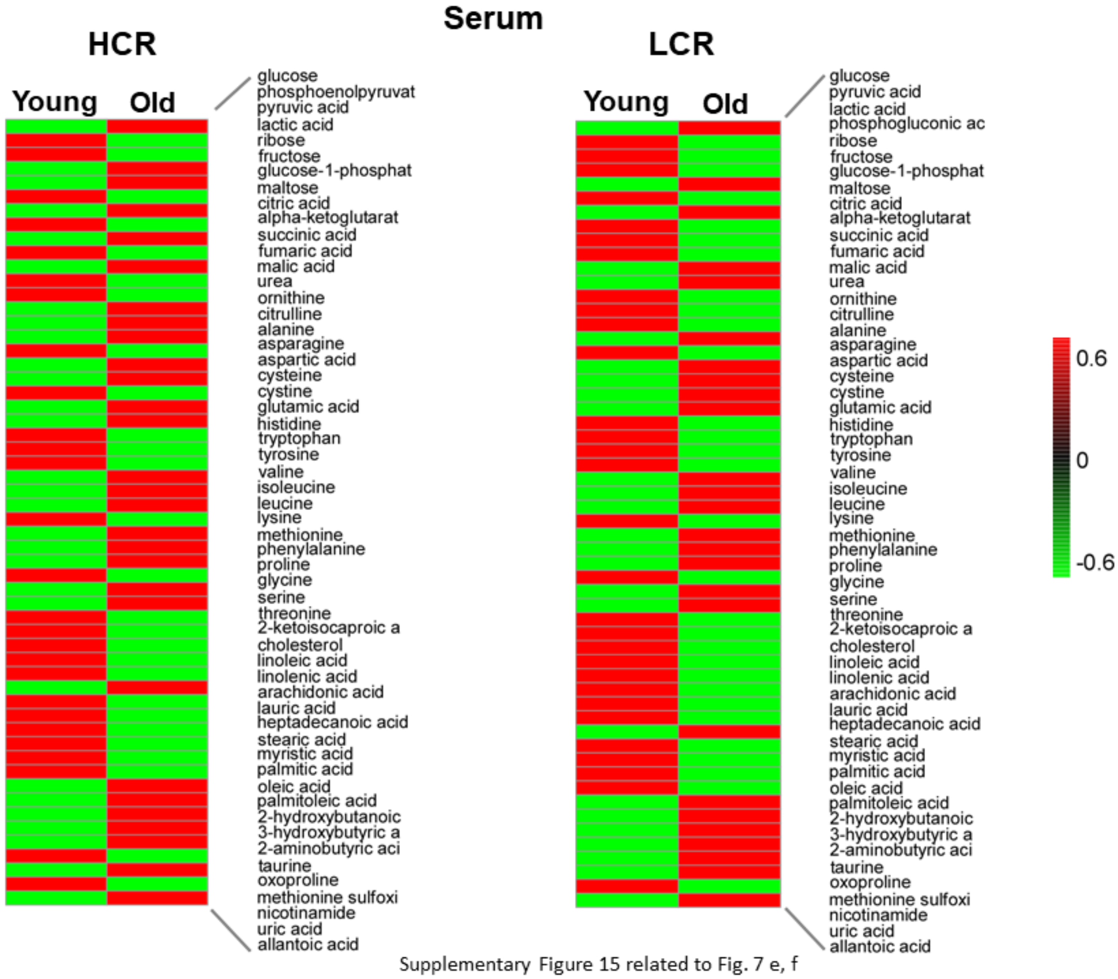


Supplementary Figure 15 related to Fig. 7 e, f

**Supplementary Fig. 15 related to Fig. 7 e, f. Comparative metabolomics of HCR *vs.* LCR, young *vs.* old, in serum**

Displayed are the average heatmaps of significantly changed metabolites in serum from HCR (left) and LCR (right) that identify the metabolites depicted in the heat maps of Figure 7 e, f.
